# Novel Distance Regression for Repeated Outcomes With Missing Data: Applications to Longitudinal and Crossover Studies of Microbiome Beta‐Diversity

**DOI:** 10.1002/sim.70654

**Published:** 2026-07-02

**Authors:** Jinyuan Liu, Ke Xu, Jane F. Ferguson, Kaidi Kang, Yue Wang, Yuqi Qiu, Lucy Shao, Shengjia Tu, Tanya T. Nguyen, Tuo Lin, Xinlian Zhang

**Affiliations:** ^1^ Department of Biostatistics Vanderbilt University Nashville Tennessee USA; ^2^ Division of Cardiovascular Medicine, Department of Medicine Vanderbilt University Nashville Tennessee USA; ^3^ Department of Biostatistics and Informatics University of Colorado Anschutz Medical Campus Aurora Colorado USA; ^4^ KLATASDS‐MOE, School of Statistics East China Normal University Shanghai China; ^5^ Division of Biostatistics and Bioinformatics UC San Diego La Jolla California USA; ^6^ Center for Microbiome Innovation UC San Diego La Jolla California USA; ^7^ Department of Psychiatry UC San Diego La Jolla California USA; ^8^ Stein Institute for Research on Aging UC San Diego La Jolla California USA; ^9^ Department of Biostatistics University of Florida Gainesville Florida USA

**Keywords:** between‐subject outcome, feature aggregation, missing at random, semiparametric inference, U‐statistics, weighted estimating equation

## Abstract

The human microbiome plays a crucial role in health, but understanding its dynamic relationship with the host requires regular monitoring. Beyond challenges such as high dimensionality and sparsity, additional complexities arise, particularly within‐cluster correlation from repeated measures and pervasive missing data. To address these issues, we develop Edger, a novel distance regression method for modeling community‐level beta‐diversity dynamics and their interactions with treatment or host physiology. By focusing on beta‐diversity, a distance metric between microbial profiles, Edger (*E*nsembled semiparametric *d*istance‐based *g*eneralized *e*stimation for *r*epeated outcomes) directly models these distances as repeated outcomes, yielding interpretable coefficients and enabling a covariate batching strategy to mitigate omitted variable bias. Our semiparametric inference framework eliminates the need for time‐consuming permutation tests, distinguishes between‐cluster heterogeneity from within‐cluster fluctuations, and allows flexible specification of working correlation structures. To handle missing data, we assume a missing‐at‐random (MAR) mechanism and incorporate a between‐subject propensity score in the repeated distance regression to provide seamless joint inference, ensuring robust variance estimation without casewise deletion. Additionally, we introduce an algorithm to generate synthetic data from real‐world microbial counts while preserving their zero‐inflated and correlated nature. Edger demonstrates superior inferential power and computational efficiency through our numerical studies and real‐world applications, making it a valuable tool for uncovering microbiome‐host interactions and advancing multi‐omics data integration.

## 1 Introduction

Compelling evidence has revealed the critical role of the gut microbiome in human diseases, ranging from diabetes to inflammatory bowel disease and from HIV infection to schizophrenia [1, 2, 3]. The human microbiome consists of myriad bacteria, viruses, and fungi that are highly dynamic, with factors such as diet, environmental exposure, and genetics influencing its composition. Microbiome data are often represented as tens of thousands of operational taxonomic units (OTUs), creating a high‐dimensional and complex analytical landscape. Recently, large‐scale longitudinal studies have been increasingly deployed to examine temporal changes in microbial compositions and their associations with clinical phenotypes or treatments [4].

As the field shifts toward longitudinal analyses, the statistical methodologies for repeated high‐throughput microbiome sequences remain underdeveloped. Beyond the inherent challenges of cross‐sectional microbiome analysis, repeated measures introduce within‐cluster correlation and missing data complications that require specialized consideration. Existing methodological developments have largely focused on microbial‐level taxa data [4, 5]. However, studies employing these methods often report weak signals with low replicability, limiting their interpretability and impact [6, 7].

On the other hand, ecological indices incorporating phylogenetic structures of the taxa, such as various diversity metrics of the microbial community, offer promising biological insights [8, 9, 10]. Many studies prioritize these composite diversity metrics, particularly the within‐subject alpha‐diversity (e.g., Shannon index) and the between‐subject beta‐diversity (e.g., Jaccard, Bray–Curtis indices) [3, 11]. While extending longitudinal models to alpha‐diversity is relatively straightforward, beta‐diversity presents unique challenges in the form of distances between subjects. Nevertheless, quite often, even when no significant changes in richness or evenness (captured by alpha‐diversity) are observed, microbial community composition can still fluctuate substantially over time [12]. Consequently, examining temporal changes in the beta‐diversity is crucial for capturing meaningful microbiome dynamics.

Existing methods, such as PERMANOVA (permutational multivariate analysis of variance) and PERMDISP (permutational multivariate analysis of dispersion), assume independent samples under the null [13, 14], making them less suitable for repeated beta‐diversity measures [15] (see Table 1). The most relevant extension, GLMM‐MiRKAT [6], incorporates mixed effects but treats beta‐diversity as a measurement error rather than an outcome, limiting its capacity to characterize the temporal trends. Moreover, GLMM‐MiRKAT relies on a single permutation‐based omnibus test, which is computationally intensive and difficult to interpret in terms of specific covariate effects. Missing data are further handled by casewise deletion, which reduces power and introduces bias when dropout is not completely at random [16].

**TABLE 1:** Comparison of beta‐diversity based methods in terms of inferential targets and hypotheses addressed.

Method	Target of inference	Hypotheses addressed
PERMANOVA	Differences in the centroid locations of multivariate microbial compositions between groups	Tests whether overall microbial composition differs between groups
PERMDISP	Differences in within‐group dispersion (heterogeneity) of beta‐diversity	Tests whether groups differ in community heterogeneity (dispersion)
GLMM‐MiRKAT	Associations between microbiome composition and host traits using kernel‐based regression	Tests whether microbiome composition is associated with host outcomes, possibly over time
Proposed Edger	Change in beta‐diversity over time, or community‐level heterogeneity dynamics	Tests time effects, group effects, and group‐by‐time interactions in community dissimilarity

To address these limitations, we propose a semiparametric distance‐regression framework that models repeated beta‐diversity directly as the outcome. Unlike kernel‐based approaches that regress host traits on microbial composition, our method treats beta‐diversity itself as the modeled response, enabling formal inference on temporal shifts and group‐by‐time interactions in microbial dissimilarity. The framework flexibly captures temporal patterns (e.g., linear, quadratic, spline, or change points) and provides interpretable effect sizes for continuous, categorical, and high‐dimensional covariates. By leveraging a distance‐based semiparametric form, it accommodates time‐varying effects, mitigates overparameterization through covariate aggregation, and remains robust under small sample sizes. Importantly, inference does not rely on permutation, ensuring computational efficiency and valid handling of missing data under realistic mechanisms [16, 17]. Although motivated by longitudinal microbiome studies, the framework generalizes naturally to other clustered data structures, making it a timely addition to the statistical toolbox for repeated‐measure analyzes in biomedical research.

The rest of the paper is organized as follows. Section 2 introduces the distance regression in cross‐sectional data before enlarging the setup to repeated distance outcomes. Section 3 discusses the semiparametric inference for complete and missing data using a between‐subject‐level weight. Section 4 first exemplifies a new data‐generating algorithm to calibrate real‐world data and then evaluates the model performance compared with existing approaches. Section 5 provides two real‐world applications; one deployed the longitudinal design to debunk the gut‐brain axis, and another evaluated the dietary intervention using a crossover design. In Section 6, we give our concluding remarks.

## 2 Distance Regression for Beta‐Diversity

### 2.1 Cross‐Sectional Data

Consider a cross‐sectional study with sample size n. Let $y_{i}={\left(y_{i}^{1},\ldots ,y_{i}^{h}\right)}^{⊤}$ denote a vector of OTU relative abundance (normalized proportions) for subject i, where the dimension h can be large (e.g., h>10,000). While direct modeling of high‐dimensional compositional microbiome data is challenging, analyzes often begin with diversity metrics that aggregate taxonomic information and relate these summaries to treatments or clinical phenotypes [14].

For within‐subject summaries, generalized linear models (GLMs) can be applied to alpha‐diversity measures, such as species richness $O_{i}=\sum_{j=1}^{h}I(y_{i}^{j}>0)$, which quantifies the number of observed taxa within an individual [3, 18]. In contrast, beta‐diversity characterizes between‐subject variation by measuring dissimilarity between pairs of microbial profiles.

Formally, each beta‐diversity outcome is defined as a distance between two subjects' microbial compositions, denoted as $f\left(y_{i}\right)=d\left(y_{i_{1}},y_{i_{2}}\right):=d_{i}$ for a pair $i=\left(i_{1},i_{2}\right)\in C_{2}^{n},$ where $C_{s}^{n}$ denotes the set of unordered s‐combinations drawn from the integer set {1,…,n}. For example, the Bray–Curtis distance quantifies dissimilarity as one minus twice the total shared abundance divided by the total abundance across the pair, taking values between 0 (identical profiles) and 1 (no shared taxa).

Because beta‐diversity is a nonlinear functional of high‐dimensional abundance vectors, it can capture complex distributional features of the microbiome, which can be difficult to detect using taxon‐wise analyses. A widely used approach for assessing associations between beta‐diversity and categorical phenotypes is the PERMANOVA, which partitions distances into within‐ and between‐group components via permutation testing [19].

However, PERMANOVA has several limitations: It relies on computationally intensive permutations, accommodates only a limited class of predictors, and offers limited flexibility for modeling continuous or high‐dimensional covariates, particularly when predictors themselves are defined at the between‐subject level (e.g., biological or genetic distances). To address these limitations, the *Ensembled distance‐based generalized estimation* framework, also referred to as Edge distance regression [10], places beta‐diversity analysis directly within a regression paradigm [20].

Let $x_{i}$ denote the explanatory variables for subject i, and let $f\left(y_{i}\right)=d_{i}$. Throughout, we distinguish notation for individual subjects (i) and unordered subject pairs (i). Edge distance regression models the beta‐diversity outcome $d_{i}$ as a function of pairwise covariates $x_{i}={\left(x_{i_{1}},x_{i_{2}}\right)}^{⊤}$ by specifying the *conditional mean*, or *restricted moment* relationship analogous to that of a GLM [21]:

(1) $$f\left(y_{i}\right)=d_{i},\;E\left\{f\left(y_{i}\right)|x_{i_{1}},x_{i_{2}};\beta \right\}=h\left(x_{i};\beta \right)=\exp\left\{\beta^{⊤}\delta \left(x_{i}\right)\right\},$$

where $\delta \left(x_{i}\right)$ is some transformation of $x_{i}$, and exp(·) accommodates the non‐negativity of distance responses.

This specification defines a *functional response* regression [22], in which the outcome is a function of multiple subjects' observations rather than a single individual response. In general, $E\left\{f\left(y_{i_{1}},\ldots ,y_{i_{s}}\right)|x_{i_{1}},\ldots ,x_{i_{s}};\beta \right\}=h\left(x_{i_{1}},\ldots ,x_{i_{s}};\beta \right),$ $\left(i_{1},\ldots ,i_{s}\right)\in C_{s}^{n}$. In our setting, s=2, corresponding to pairwise distances. Inference targets the finite‐dimensional parameter β, while leaving the distribution of the beta‐diversity outcome $d_{i}$ unspecified. This semiparametric setup offers robustness to distributional misspecification, an important consideration given the non‐Gaussian and sparsely correlated structure of beta‐diversity measures, while retaining asymptotic optimality under standard regularity conditions [23, 24, 25, 26].

To elaborate on (1), consider comparing the beta‐diversity between two groups ($x_{i}\in \{\text{H,D}\}$, healthy vs. disease). Applying one‐hot encoding to $x_{i}={\left(x_{i_{1}},x_{i_{2}}\right)}^{⊤}$ yields the vector of between‐subject dummy variables:

(2) $$\begin{array}{cc} \delta (x_{i}) & ={\left(\delta_{\text{HH}}\left(x_{i}\right),\delta_{\text{DD}}\left(x_{i}\right),\delta_{\text{DH}}\left(x_{i}\right)\right)}^{⊤},\;\delta (\cdot ):\{\text{H,D}\}\\ & \;\times \{\text{H,D}\}\mapsto {\left\{0,1\right\}}^{3}. \end{array}$$

which partitions all subject pairs into healthy–healthy (HH), disease–disease (DD), and mixed disease–healthy (DH) pairs. Taking HH pairs as the reference group, the distance model becomes $h\left(x_{i};\beta \right)=\exp\left\{\beta_{0}+\beta_{\text{DD}}\delta_{\text{DD}}\left(x_{i}\right)+\beta_{\text{DH}}\delta_{\text{DH}}\left(x_{i}\right)\right\}$ in (1).

A key advantage of this distance regression is that the coefficients β can delineate the granular patterns of heterogeneity in the high‐dimensional microbiome through pairwise distances $d_{i}$, which is difficult using conventional approaches. For example, this specification has been used to separate dispersion and location effects in microbiome beta‐diversity when comparing patients with alcoholic liver disease to healthy controls [18, 20]. In this context, $\beta_{\text{DD}}$ captures within‐disease dispersion (scale) relative to the healthy reference, whereas $\beta_{\text{DH}}$ reflects systematic between‐group (location) differences. When both effects are present, their relative magnitudes indicate whether observed dissimilarities arise mainly from increased within‐group variability, systematic group shifts, or a combination of the two.

### 2.2 Repeated Outcomes

Motivated by the increasing prevalence of longitudinal microbiome studies, we extend the cross‐sectional distance regression model in (1) to accommodate repeated pairwise beta‐diversity outcomes. Throughout, we use *visit*, *time*, and *assessment* interchangeably, depending on the context.

Importantly, this extension does not attempt to reconstruct or model a single cross‐time beta‐diversity matrix spanning all subjects and all assessment points. Instead, beta‐diversity at each visit is treated as a well‐defined composite outcome computed from contemporaneous microbial compositions, and we explicitly model how this dissimilarity evolves over time or differs with and without an intervention. Accordingly, the primary target of inference is the *change in beta‐diversity over time* that reflects shifts in overall community structure, rather than the taxon‐specific longitudinal changes within subjects.

#### 2.2.1 Group Comparisons Over Time

Suppose each subject is measured at m assessments indexed by $t\in C_{1}^{m}$, yielding the OTU relative abundances $y_{it}$. For a subject pair $i=\left(i_{1},i_{2}\right)$, the visit‐specific beta‐diversity $f\left(y_{it}\right)=d\left(y_{i_{1}t},y_{i_{2}t}\right)$ can be computed whenever both subjects are observed at visit t. In typical applications, such as treatment or clinical phenotype comparisons, predictors include *time*, *group*, and their *interaction*. We denote the (time‐invariant) group indicator by $x_{i}\in C_{1}^{K}$; extensions to time‐varying covariates are discussed in Section 2.2.2.

To distinguish baseline differences from longitudinal dynamics, we decompose the model into baseline and post‐baseline components. The baseline component captures cross‐sectional differences in beta‐diversity at study entry, while the post‐baseline component characterizes temporal evolution and group‐specific trajectories over repeated assessments. Specifically, we define the baseline component as $\phi_{0}(x_{i};\beta_{0},\beta_{G})=\beta_{0}+\beta_{G}^{⊤}\delta (x_{i}),$ which describes the mean *baseline* beta‐diversity (on the log scale) within each pairwise group indexed by the between‐subject indicators $\delta (x_{i})$ as in (2).

Under this decomposition, the unified *Edger* framework (*E*nsembled semiparametric *d*istance‐based *g*eneralized *e*stimation for *r*epeated outcomes) models the conditional mean of pairwise beta‐diversity as

(3) $$\begin{array}{cc} & E\left\{f\left(y_{it}\right)|\delta (x_{i}),t;\beta \right\}\\ & \;=\exp\left\{\phi_{0}(x_{i};\beta_{0},\beta_{G})+F{\left(t\right)}^{⊤}\phi_{1}(x_{i};\beta_{T},\beta_{I})\right\}, \end{array}$$

where F(t) denotes an analyst‐defined function of time, chosen to flexibly represent temporal structure (e.g., factor, linear, quadratic, or spline basis), and $\phi_{1}(x_{i};\beta_{T},\beta_{I})$ collects the post‐baseline parameters, including overall time effects ($\beta_{T}$) and group‐by‐time interaction effects ($\beta_{I}$).

Biologically, the coefficients in (3) admit direct interpretations in terms of community‐level dissimilarity. The baseline component $\phi_{0}(\cdot )$ reflects cross‐sectional differences in mean beta‐diversity: positive group coefficients $\beta_{G}$ indicate greater between‐subject heterogeneity, whereas negative values indicate increased similarity or community convergence. The post‐baseline terms describe how this mean dissimilarity evolves over time, enabling inference on community divergence (e.g., $\beta_{I}>0$), convergence (e.g., $\beta_{I}<0$), or temporal stability (e.g., $\beta_{T}=\beta_{I}=0$) [27, 28].

Different choices of F(t) also allow the model to accommodate a wide range of temporal patterns. For example, F(t)=t specifies linear trajectories, with group‐specific slopes captured through interaction terms; quadratic bases allow nonlinear trends; and spline bases provide additional flexibility for irregular or complex temporal dynamics [29]. In the following, we illustrate several common modeling scenarios.

##### 2.2.1.1 Example 1: Longitudinal Response Profiles

First, by treating time as an m×1 indicator I(t), the model imposes no parametric constraints on the underlying temporal trajectory. Under this saturated specification, group‐specific patterns of mean beta‐diversity over time are described by

(4) $$\begin{array}{cc} & E\left\{f\left(y_{it}\right)|\delta (x_{i}),t;\beta \right\}\\ & \;=\exp\left\{\beta_{0}+\beta_{1}^{⊤}I(t)+\beta_{2}^{⊤}\delta (x_{i})+\beta_{3}^{⊤}\varpi (x_{i},t)\right\}, \end{array}$$

where each entry of I(t) is $I_{it^{\prime }}\left(t\right)=1$ if $t=t^{\prime }$ and 0 otherwise. Extending the group encoding in (2), for K groups we define $\delta (x_{i})={\left(\delta_{12}\left(x_{i}\right),\ldots ,\delta_{KK}\left(x_{i}\right)\right)}^{⊤}$, where $\delta_{k_{1}k_{2}}\left(x_{i}\right)=1$ if $x_{i}=\left\{x_{i_{1}},x_{i_{2}}\right\}=\left\{k_{1},k_{2}\right\}$ with $1\leq k_{1}\leq k_{2}\leq K$ (to ensure a unique representation of unordered pairs). The interaction term $\varpi (x_{i},t)$ then collects group‐by‐time indicators, with corresponding coefficients $\beta_{3}$ that are typically of primary interest.

Inference may proceed by first testing an omnibus group‐by‐time interaction. If this interaction is not significant, it can be removed and the model refit to assess main effects. More generally, hypotheses of the form $H_{0}:C\beta =0$ versus $H_{a}:C\beta \neq 0$, can be tested using Wald statistics with an appropriate contrast matrix C (see Section 3.2.3).

As discussed in Section 2.2.1, the coefficients in (4) admit direct interpretations in terms of cross‐sectional heterogeneity and group‐specific longitudinal changes in community‐level dissimilarity. To illustrate, consider a binary group variable $x_{i}\in \{$ H,D} observed over three visits. Using one‐hot encoding with HH pairs $\delta_{\text{HH}}\left(x_{i}\right)$ as the reference, the intercept $\beta_{0}$ represents the mean baseline beta‐diversity (on the log scale) among healthy pairs. The temporal coefficients $\beta_{1}={\left(\beta_{t2},\beta_{t3}\right)}^{⊤}$ characterize changes in beta‐diversity for this reference group at visits 2 and 3. The main group effects $\beta_{2}={\left(\beta_{\text{DH}},\beta_{\text{DD}}\right)}^{⊤}$ capture baseline differences for mixed and disease pairs relative to healthy pairs, while the interaction terms $\beta_{3}={\left(\beta_{t2,\text{DH}},\beta_{t2,\text{DD}},\beta_{t3,\text{DH}},\beta_{t3,\text{DD}}\right)}^{⊤}$ quantify group‐specific longitudinal effects, indicating whether temporal changes in location (mixed DH pairs) and dispersion (DD, or DD pairs) diverge from the healthy reference over time.

##### 2.2.1.2 Example 2: Longitudinal Change‐Point Model

When a larger number of assessments is available, one may posit some form to describe the trajectories of beta‐diversity. For example, community dynamics may evolve approximately linearly up to a known critical time point $t^{*}$, followed by accelerated divergence or stabilization thereafter. The proposed Edger framework accommodates such behavior by incorporating a change‐point structure [17].

Specifically, define ${\left(t-t^{*}\right)}_{+}=\max\{0,(t-t^{*})\}$ and consider the model

(5) $$\begin{array}{cc} & E\left\{f\left(y_{it}\right)|\delta (x_{i}),\;t\right\}\\ & \;=\exp\left\{\beta_{0}+\beta_{1}t+\beta_{2}{\left(t-t^{*}\right)}_{+}+\beta_{3}{\left(t-t^{*}\right)}_{+}^{2}+\beta_{4}^{⊤}\delta (x_{i})\right\}. \end{array}$$

Under this specification, $\beta_{1}$ governs the pre‐change‐point trend, while $\beta_{2}$ and $\beta_{3}$ capture deviations in slope and curvature after $t^{*}$.

The presence of such a structural change can be formally assessed by testing the joint null hypothesis $\beta_{2}=\beta_{3}=0$. Rejection of this null provides evidence for a qualitative shift in community‐level dissimilarity dynamics, consistent with a transition from relative stability to accelerated divergence or convergence, and may reflect ecological reorganization associated with intervention onset, disease progression, or environmental perturbations [27].

##### 2.2.1.3 Example 3: Two‐by‐two Crossover Design

The Edger framework in (4) naturally extends to crossover designs, which are commonly used to evaluate treatment, period, and carryover effects [30]. Consider a two‐by‐two crossover study comparing treatment and control arms, with treatment assignment $x_{it}\in \{0,1\}$ over two periods (t=1,2). Pairwise group membership at each period is encoded via the between‐subject indicators $\delta (x_{it})={\left(\delta_{11}\left(x_{it}\right),\delta_{00}\left(x_{it}\right),\delta_{01}\left(x_{it}\right)\right)}^{⊤}$, corresponding to treated–treated, control–control, and mixed pairs, respectively.

Following standard crossover analysis, inference proceeds by first testing the group‐by‐time interaction to assess potential carryover effects. In the absence of carryover, the interaction is removed and the main‐effects model is refit to interpret the treatment effects [17]. Under this reduced model, beta‐diversity in the first period is given by

(6) $$E\left\{f\left(y_{i1}\right)|\delta (x_{i1});\beta \right\}=\exp\left\{\beta_{0}+\beta_{11}\delta_{11}\left(x_{i1}\right)+\beta_{01}\delta_{01}\left(x_{i1}\right)\right\}.$$

In the second period, after treatment switching,

(7) $$\begin{array}{cc} & E\left\{f\left(y_{i2}\right)|\delta (x_{i2});\beta \right\}\\ & \;=\exp\left\{\left(\beta_{0}+\beta_{t}\right)+\beta_{11}\delta_{11}\left(x_{i2}\right)+\beta_{01}\delta_{01}\left(x_{i2}\right)\right\}, \end{array}$$

where $\beta_{t}$ is the period effect and $\left\{\beta_{11},\beta_{01}\right\}$ are treatment‐related parameters assumed constant across periods.

If the treatment has no effect, microbial compositions are similar across arms and the mean beta‐diversity is equal across all pair types. When a treatment effect is present, Edger distinguishes two mechanisms (see also Figure S1 in the Supporting Information):

- *Dispersional treatment effect*: A significant $\beta_{11}$ indicates that treatment alters within‐arm variability, reflected by changes in dissimilarity among treated pairs relative to controls.
- *Locational treatment effect*: A significant $\beta_{01}$ indicates systematic distributional shifts between treatment and control arms [31] (e.g., left‐skewed OTU distributions among treated subjects and right‐skewed among controls while their within‐group variability remains comparable).

The direction of these effects is also biologically informative. Negative values of $\beta_{11}$ or $\beta_{01}$ correspond to increased microbial similarity relative to the control reference, consistent with treatment‐induced convergence or stabilization. In contrast, positive values reflect greater between‐subject dissimilarity, suggesting heterogeneous or individualized microbial responses, consistent with ecological divergence or host‐specific effects [27, 28].

#### 2.2.2 Account for Covariates

The unified form in (3) readily allows for time‐varying covariates, which oftentimes are the key contributors to altering the microbial composition. We allow different variable types (categorical, continuous, high‐dimensional) to be handled separately when constructing their between‐subject counterparts.

Categorical covariates can be encoded similarly as in (2). Denote by $w_{it}$ the additional covariate for the ith subject (with K levels) and $w_{it}={\left(w_{i_{1}t},w_{i_{2}t}\right)}^{⊤}$ for the ith pair, by applying the one‐hot encoding, we obtain the t‐specific pairwise dummy variables $\delta (w_{it})\in {\mathbb{R}}^{K+C_{2}^{K}}$ suitable for (4).

For a continuous covariate $z_{it},$ one can map each pairwise observation $\left\{z_{i_{1}t},z_{i_{2}t}\right\}$ into a between‐subject attribute using some function $g(\cdot ):\mathbb{R}\times \mathbb{R}\mapsto {\mathbb{R}}_{\geq 0}$ (e.g., $L_{2}$ distance). Further, the study may encounter high‐dimensionality with Q covariates and Q>>n. Here, instead of constructing separate effects, the distance formulation allows for a certain level of aggregation. Specifically, one can construct an ensembled covariate by aggregating all covariates at tth visit into one distance as $g\left(z_{it}\right)=g(z_{i_{1}t}^{Q\times 1},z_{i_{2}t}^{Q\times 1})$, which is useful, for instance, in microbiome studies that also, control for time‐varying metabolomic sequences [32]. Also, the covariates can be batched to elevate scientific relevance per domain knowledge, especially if they follow a tree structure or latent construct [33]. One example is the clinical instrument of cognitive functioning [34], which spans across six domains such as “Memory” and “Attention,” then respective subdomain distances can be constructed to increase the model interpretability.

Next, after transforming the covariates into between‐subject‐level metrics, we adjust for them directly in the main group model as:

(8) $$\begin{array}{cc} & E\left\{f\left(y_{it}\right)|q_{it}\right\}\\ & \;=\exp\left\{\phi_{0}(x_{i};\beta_{G})+F{\left(t\right)}^{⊤}\phi_{1}(x_{i};\beta_{T},\beta_{I})+\eta^{⊤}\delta (w_{it})+\xi^{⊤}g\left(z_{it}\right)\right\}, \end{array}$$

where $q_{it}={\left(\delta {\left(x_{i}\right)}^{⊤},\delta {\left(w_{it}\right)}^{⊤},g{\left(z_{it}\right)}^{⊤}\right)}^{⊤}$ and {η,ξ} are additional parameters for covariates. The aggregated continuous covariate vector $g\left(z_{it}\right)$ has dimension $Q^{\prime }$ (≤Q), which controls the resolution of batching. This aggregation can be viewed as a form of covariate dimension reduction and may be determined based on subject‐matter expertise or selected via cross‐validation [10].

Notably, many longitudinal human microbiome studies involve a large number of covariates but relatively limited sample sizes [3]. In such settings, dimension‐reduction techniques such as principal component analysis (PCA) are commonly applied as a preprocessing step [35]. However, adjustment based solely on the leading principal components can lead to substantial information loss, which may in turn propagate bias in downstream inference [36], as demonstrated in our simulations (see Section 4.2.1).

Instead, our distance regression framework provides an alternative by aggregating or batching covariates to mitigate potential bias [37]. More importantly, it enables the integration of multi‐omics data through a multi‐modality distance regression framework for repeated measures (e.g., when $z_{it}$ represents other sources of ‐omics data), by flexibly accommodating heterogeneous feature dimensions across ‐omic layers using biological distances [38, 39].

## 3 Semiparametric Inference

To tackle the inferential challenge for the non‐i.i.d. pairwise outcomes, the semiparametric approach requesting no distributional assumption becomes a natural choice [21, 24]. In the cross‐sectional case, a class of *univariate U‐statistics‐based generalized estimating equations* (UGEE) has been deployed to address the correlations among $f\left(y_{i}\right)$ [20, 26]. Distinct from conventional within‐subject estimating equations, these modeling units are non‐independent pairs; for example, the quantities formed by two pairs i=(1,2) and $i^{\prime }=(1,3)$ correlate as they share the same information from the first subject. This type of *between‐pair* correlations are induced by overlapping subjects; yet by incorporating the U‐statistics into the estimating equation, one can readily apply the Hájek projection [26] to break their interlocking dependencies so that the central limit theorem (CLT) applies for the inference.

### 3.1 Complete Data

Aside from the previous between‐pair correlations, repeated beta‐diversity outcomes induce another layer of *within‐pair* (akin to the within‐subject) correlations that we need to address in the inference. Start with the complete data case; in what follows, we simplify the notation in (8) by denoting $f\left(y_{it}\right)=f_{i,t}$ the beta‐diversity and $h\left(q_{it};\beta \right)=h_{i,t}\left(\beta \right)$ the smoothed function of explanatory variables for the ith pair at time t. Let $f_{i}={\left(f_{i,1},\ldots ,f_{i,m}\right)}^{⊤}$ and $h_{i}\left(\beta \right)={\left(h_{i,1},\ldots ,h_{i,m}\right)}^{⊤}$, we denote their differences by $S_{i}^{m\times 1}\left(\beta \right)=f_{i}-h_{i}\left(\beta \right)$ and define the multivariate UGEE:

(9) $$U_{n}\left(\beta \right)=\underset{i\in C_{2}^{n}}{\sum }U_{n,i}\left(\beta \right)=\underset{i\in C_{2}^{n}}{\sum }D_{i}V_{i}^{-1}S_{i}\left(\beta \right)=0.$$

Distinct from the univariate UGEE [20], here the partial derivative $D_{i}=(\partial /\partial \beta )S_{i}\left(\beta \right)$ is a p×m matrix incorporating the time dimension (m). Naturally, (9) becomes suitable for repeated measures by allowing a working variance–covariance matrix for the unknown $V_{i}$. For example, denote by $V_{i,t}=Var\left(f_{i,t}|q_{it}\right)$ the conditional variance of each repeated beta‐diversity $f_{i,t}$, we can specify the working variance–covariance matrix as

(10) $$V_{i}=A_{i}^{\left(1/2\right)}R\left(\varphi \right)A_{i}^{\left(1/2\right)},\;A_{i}=diag_{1\leq t\leq m}\left(V_{i,t}\right),$$

where the *working correlation* $R{\left(\varphi \right)}^{m\times m}$ captures the added layer of within‐pair, or within‐cluster correlations among repeated pairwise outcomes. Akin to the GEE, adopting the working variance leads to valid inference for β, albeit our uncertainty about the assumed forms for $V_{i,t}$ or R(φ).

Henceforth, the dependencies are decomposed into between‐pair and within‐pair correlations, which are jointly handled within the multivariate UGEE. The between‐pair correlations are accounted for through the U‐statistics [40], while the within‐pair temporal correlations are accommodated via the working covariance in (10) and the sandwich variance estimation [20]. This construction yields a consistent and asymptotically normal estimator $\hat{\beta }$ [26], whose semiparametric efficiency can be established by availing the equivalence classes in the Hilbert tangent space. Although beyond the scope of this paper, $U_{n,i}\left(\beta \right)$ in (9) is shown to be the efficient score, which yields the estimator with minimal variance under complete data [21, 36, 41].

Moreover, while microbial abundance data are often sparse and zero‐inflated, UGEE operates on beta‐diversity distances, which are continuous, bounded measures of compositional dissimilarity, thus mitigating the direct impact of sparsity and zero‐inflation. In practice, the parameter φ in (10) is unknown, but the asymptotic normality of $\hat{\beta }$ is guaranteed if a $\sqrt{n}$‐consistent estimator is substituted [24]. In the presence of time‐varying covariates, the working independent correlation may be used, with R(φ) reducing to an identity matrix and assured asymptotic normality of $\hat{\beta }$, while trading off some efficiency. Nonetheless, modeling within‐pair temporal correlation can yield certain efficiency gains, consistent with the GEE [17].

### 3.2 Missing Data

Missing data is common in longitudinal microbiome studies, and we focus on study dropout that is most prevalent [17]. Unlike many other microbiome data maltreated with complete‐case analyses [6], we consider more realistic missing mechanisms to help mitigate biases by availing all available information. Throughout, the covariates are assumed complete and the OTU abundance $y_{it}$ is subject to missing [42].

#### 3.2.1 Notations and Assumptions

We index by $R_{it}=1$ if $y_{it}$ is observed, $R_{it}=0$ if $y_{it}$ is missing. The assumptions are made for the subject‐level i.i.d. data as in conventional settings.

*A1*—*Monotone missing data pattern* (MMDP): if the dropout occurs at t, the subsequent observations of $y_{it}$ are missing. The *full outcome* can thus be partitioned into $\left\{y_{it}^{O},y_{it}^{M}\right\},$ with $y_{it}^{O}=\{y_{is};s\in C_{1}^{\left(t-1\right)}\}$ the collections of *observed outcomes* if this subject drops out at t.

*A2*—*Missing at random* (MAR): the probability of observing $y_{it}$ at time t ($R_{it}=1$) depends only on the prior outcomes $y_{it}^{O}$ and explanatory variables $q_{i}=\{q_{is};s\in C_{1}^{\left(t-1\right)}\}$:

(11) $$\pi_{it}=\mathrm{Pr}(R_{it}=1|y_{i},q_{i})\overset{\text{MAR}}{=}\mathrm{Pr}(R_{it}=1|y_{it}^{O},q_{i})=\pi (y_{it}^{O},q_{i}).$$

*A3*—No missing data at baseline t=1: $R_{i1}\equiv \pi_{i1}\equiv 1$ for all 1≤i≤n.

Our models focus on the pairs, note that the between‐subject response $f_{i,t}=d\left(y_{i_{1}t},y_{i_{2}t}\right)$ can be calculated only when both $y_{i_{1}t}$ and $y_{i_{2}t}$ are observed, that is, when $R_{i_{1}t}=R_{i_{2}t}=1$. Therefore, the complete case index for the ith pair is $R_{i,t}=R_{i_{1}t}R_{i_{2}t}$, upon which we define the *pairwise propensity score*:

(12) $$\pi_{i,t}=\pi (y_{i_{1}t}^{O},q_{i_{1}})\pi (y_{i_{2}t}^{O},q_{i_{2}})=\mathrm{Pr}\left(R_{i,t}=1|y_{i_{1}t}^{O},y_{i_{2}t}^{O},q_{i_{1}},q_{i_{2}}\right).$$

Accordingly, the independence among subjects implies that the MAR in A2 carries over to pairs, allowing us to extend the *weighting* technique [16] to pairwise outcomes.

#### 3.2.2 Between‐Subject Inverse Probability Weighting (BIPW)

If the complete‐case probability $\pi_{it}$ in (11) is known and free of parameters (e.g., missing by design), then $\pi_{i,t}$ in (12) can be easily calculated. With missing data, the observed pairs are no longer representative, and we inflate their contributions to rebalance the selection bias [16]. To this end, we define the inverse weights $\phi_{i,t}:=R_{i,t}/\pi_{i,t}$ for pairs and incorporate them in the multivariate UGEE in (9), which leads to the *Weighted U‐statistics‐based generalized estimating equations* (Weighted UGEE):

(13) $$\begin{array}{cc} U_{n}\left(\beta \right) & =\underset{i\in C_{2}^{n}}{\sum }U_{n,i}\left(\beta \right)=\underset{i\in C_{2}^{n}}{\sum }D_{i}V_{i}^{-1}\Delta_{i}S_{i}\left(\beta \right)=0,\\ \Delta_{i} & =diag_{1\leq t\leq m}\left(\phi_{i,t}\right), \end{array}$$

where $S_{i}\left(\beta \right)$, $D_{i}$ and $V_{i}$ remain the definition as in Section 3.1.

More prevalently, the probability $\pi_{it}$ or $\pi_{i,t}$ is unknown and should be estimated. Under the MAR assumption A2, missing the outcome at time t is attributable only to the prior observations $\left\{y_{it}^{O},q_{i}\right\}$. Although it is tempting to posit a logistic regression to model $\pi_{it}$ for each subject [16, 24], in our context, this involves using all prior OTUs $y_{it}^{O}=\{y_{is};s\in C_{1}^{\left(t-1\right)}\}$ as high‐dimensional predictors and potentially leads to overfitting and non‐convergence [43].

Instead, we propose a *between‐subject inverse probability weighting* (BIPW) technique to directly model the propensity score for a pair $\pi_{i,t}$ in (12) using a regression. First, for each visit s, define $f_{is}^{O}$ as collections of all prior beta‐diversity metrics computed from observed OTUs. With the induced MAR among pairs, we model its *one‐step* transition probability from the visit (s−1) to s (2≤s≤t) with a between‐subject regression for binary pairwise outcomes [10]:

(14) $$\begin{array}{cc} & \mathrm{Pr}\left(R_{i,s}=1|R_{i,\left(s-1\right)}=1,f_{is}^{O},q_{i}\right)\\ & \;=\text{expit}\left(\gamma_{0,s}+\gamma_{f,s}^{⊤}f_{is}^{O}+\gamma_{q,s}^{⊤}q_{i}\right):=p_{i,s}(\gamma_{s}). \end{array}$$

where $q_{i}=\{q_{i_{1}},q_{i_{2}}\}$ are the (observed) covariates of the pair. By predicting $p_{i,s}(\gamma_{s})$ using the beta‐diversity that aggregates the high‐dimensional OTUs, this proposed strategy can mitigate the potential omitted variable bias [37].

Further by assumption A1, multiplying all transition probabilities $p_{i,s}(\gamma_{s})$ prior to t yields the between‐subject propensity score $\pi_{i,t}\left(\gamma \right)$ and weights $\phi_{i,t}\left(\gamma \right)$:

(15) $$\begin{array}{cc} \pi_{i,t}\left(\gamma \right) & :=\overset{t}{\underset{s=2}{\prod }}p_{i,s}\left(\gamma_{s}\right),\;\phi_{i,t}\left(\gamma \right)\\ & :=\frac{R_{i,t}}{\pi_{i,t}\left(\gamma \right)},\;\gamma ={\left(\gamma_{2}^{⊤},\ldots ,\gamma_{m}^{⊤}\right)}^{⊤}, \end{array}$$

and we specify $\Delta_{i}\left(\gamma \right)=diag_{1\leq t\leq m}\left\{\phi_{i,t}\left(\gamma \right)\right\}$ as the new diagonal weight matrix in (13).

In practice, a two‐step procedure can be adopted to estimate $\hat{\gamma }$ in (14) first and substitute $\Delta_{i}\left(\hat{\gamma }\right)$ in (13) before estimating β. To properly account for the variability of estimating γ in the first step, variance adjustment formula for $\hat{\beta }$ has been proposed [24], but the tedious formula hinders its wide usage; oftentimes, investigators completely ignore the induced variability in the first step. To resolve the issue, we introduce a joint optimization strategy to facilitate seamless and automatic adjustment without additional steps.

#### 3.2.3 Joint Inference

We term the model for the repeated beta‐diversity $f_{i,t}$ (1≤t≤m) as the *main response*module, and the model of the one‐step probabilities $p_{i,s}$ (2≤s≤t) in (14) as the *missing data*module. The joint estimating equations combine the two modules by redefining:

(16) $$\begin{array}{ccc} q_{i,t} & =\left\{\begin{array}{l} f_{i,t},\\ R_{i,\left(t-m+1\right)}, \end{array}\right. & \\ g_{i,t}\left(\theta \right) & =\left\{\begin{array}{ll} h_{i,t}\left(\beta \right), & \text{if}\;1\leq t\leq m,\\ p_{i,\left(t-m+1\right)}\left(\gamma_{t-m+1}\right), & \text{if}\;m+1\leq t\leq 2m-1, \end{array}\right.\; & \end{array}$$

where $\theta ={\left(\beta^{⊤},\gamma^{⊤}\right)}^{⊤}$. Denote by $q_{i}$ and $g_{i}(\theta )$ their respective vector versions and $S_{i}(\theta )=q_{i}-g_{i}(\theta )$ as their difference (theoretical residual). Let $G_{i}=(\partial /\partial \theta )S_{i}(\theta )$, the previous Weighted UGEE can be expanded as

(17) $$\begin{array}{cc} W_{n}\left(\theta \right) & =\underset{i\in C_{2}^{n}}{\sum }W_{n,i}\left(\theta \right)=\underset{i\in C_{2}^{n}}{\sum }G_{i}V_{i}^{-1}\Delta_{i}(\gamma )S_{i}(\theta )=0. \end{array}$$

Accordingly, $V_{i}=diag(V_{i,11},V_{i,22})$ is modified to be a *block diagonal* matrix, where $V_{i,11}$ is the working variance for the main response as in (10), and $V_{i,22}=diag_{2\leq t\leq m}\left\{p_{i,t}\left(1-p_{i,t}\right)\right\}$ reflects the variance of the binary $R_{i,t}$ in the missing data module. Also, the new weight matrix becomes

(18) $$\begin{array}{cc} \Delta_{i}(\gamma ) & =diag(\Delta_{i,11}(\gamma ),\Delta_{i,22}),\;\Delta_{i,11}(\gamma )\\ & =diag_{1\leq t\leq m}\left\{\phi_{i,t}(\gamma )\right\},\;\Delta_{i,22}=I_{\left(m-1\right)}\text{,} \end{array}$$

where $\Delta_{i,11}(\gamma )$ resembles the weights for the main response as appeared in (15), and the identity matrix $\Delta_{i,22}$ indicates no additional rebalance is needed for estimating the weights.

The estimator $\hat{\theta }$ can be obtained from Newton's method, which is guaranteed to be consistent by verifying that $E\left\{W_{n}\left(\theta \right)\right\}=0$. We summarize their asymptotic properties below and provide proof in Section S1 of the Supporting Information.

Theorem 1 (Consistency and asymptotic normality) *Let* $\hat{\theta }$ *denote the Weighted UGEE estimators from solving the joint estimating equations in* (17) *upon substituting some estimators* $\hat{\varphi }$ *for the working correlation. Applying Hájek projection to* $W_{n,i}\left(\theta \right)$ *yields*

(19) $$\begin{array}{cc} v_{i_{1}} & =E\left(W_{n,i}\left(\theta \right)|y_{i_{1}},q_{i_{1}}\right),\;\sum_{W}=4Var\left(v_{i_{1}}\right),\\ B & =E\left(\frac{\partial }{\partial \theta }W_{n,i}\right),\;\sum_{\theta }=B^{-1}\sum_{W}B^{-1}. \end{array}$$

*Then, under mild regularity conditions*, $\hat{\theta }$ *is consistent; Provided* $\hat{\varphi }$ *is* $\sqrt{n}$ *‐consistent*, $\hat{\theta }$ *is asymptotically normal* (*AN*) *with* $\sqrt{n}\left(\hat{\theta }-\theta \right)\to_{d}N\left(0,\sum_{\theta }\right)$.

A consistent sandwich variance estimator can be obtained by substituting consistent estimators of θ and moments of the respective quantities in $\sum_{\theta }$. Usually, β in the main response module is of inferential interest, partitioning $\sum_{\theta }$ in (19) yields

(20) $$\sum_{\theta }=\left(\begin{array}{cc} \sum_{\beta } & \sum_{\beta \gamma }\\ \sum_{\gamma \beta } & \sum_{\gamma } \end{array}\right),$$

which implies that$\sqrt{n}\left(\hat{\beta }-\beta \right)\to_{d}N\left(0,\sum_{\beta }\right)$ and enables hypothesis tests about β through linear contrast matrix C (with rank c). Under the null $H_{0}:C\beta =0$, we construct the Wald statistic that follows an asymptotic central $\chi^{2}$ with c degrees of freedom:

$$W_{n}=n{\left(C\hat{\beta }\right)}^{⊤}{\left(C{\hat{\sum }}_{\beta }C^{⊤}\right)}^{-1}\left(C\hat{\beta }\right)\to_{d}\chi_{c}^{2}.$$

For example, in Section 2.2.1.1 when testing the interaction terms in (4) with a three‐level pairwise group and three visits, we have $W_{n}$ $\to_{d}\chi_{4}^{2}$ (c =(3−1)×(3−1)=4).

Here, Theorem 1 applies to general working correlations R(φ), and the robust semiparametric inference ensures the asymptotic normality of $\hat{\theta }$ (and $\hat{\beta }$), even under an incorrectly‐specified working variance. This level of flexibility is especially preferable for longitudinal pairwise observations since validating their complex correlation structures is impractical.

Also, (20) implies that the variance of $\hat{\beta }$ obtained by ignoring the sampling variability of $\hat{\gamma }$ in the conventional two‐step procedure is, in fact, the variance of the conditional distribution of $\hat{\beta }$ given $\hat{\gamma }$ (i.e., $\sum_{\beta }-\sum_{\beta \gamma }\sum_{\gamma }^{-1}\sum_{\gamma \beta }$), which is smaller than its marginal variance $\sum_{\beta }$ unless $\sum_{\beta \gamma }=0$, namely, when $\hat{\beta }$ and $\hat{\gamma }$ are asymptotically uncorrelated. This condition rarely holds unless the score for β is orthogonal to an infinite‐dimensional tangent space spanned by γ, such that the limiting distribution of $\hat{\beta }$ will not be strongly affected by the precision in estimating γ [26, 36].

Therefore, with convenient and automatic variance adjustment, our joint inference procedure ensures the appropriate asymptotic behavior of $\hat{\beta }$ even in the presence of missing data, which is crucial to derive valid scientific insights.

## 4 Numerical Studies

We conduct simulation studies to demonstrate the validity (consistency and asymptotic normality) of the proposed semiparametric distance regression and its superiority over existing approaches. The advantage of covariate aggregation offered by the distance formulation was also examined. The Monte Carlo (MC) size was set to R=1000 in all model‐based simulations.

### 4.1 Validity

#### 4.1.1 Data Generation Using eCDF and Copula

To best reflect the model performance in practice, we adopted a novel way to simulate from real‐world microbiome data, which can preserve the zero‐inflated and correlated OTU features [20, 44]. Recall that the probability integral transformation of a random variable Y, denoted by U=F(y)=Pr(Y≤y), follows a uniform distribution U(0,1). By Sklar's theorem, the joint distribution function (CDF) of a random vector $Y^{h\times 1}$ can be written as h uniform marginals and a *Copula* to describe their dependence structure [45]:

(21) $$F\left(y\right)=\mathrm{Pr}\left(Y^{1}\leq y^{1},Y^{2}\leq y^{2},\ldots ,Y^{h}\leq y^{h}\right)=C\left(u^{1},u^{2},\ldots ,u^{h}\right),$$

where $U^{j}=F_{j}\left(y^{j}\right)$ is the uniform marginal and C(·) is the Copula. When the true CDFs $F_{j}\left(\cdot \right)$ are unknown, the empirical versions $U_{n}^{j}=F_{n}\left(y^{j}\right)=(1/n)\sum_{i=1}^{n}I\left(Y_{i}^{j}\leq y^{j}\right)$ can be substituted [20].

For our purpose, the empirical distributions (eCDF) of the real‐world OTU counts were first calibrated, whose feature correlations are preserved via the Copula. Next, by conducting inverse transform sampling through the constructed Copula, we obtained the simulated OTUs admitting the same joint distribution as the observed OTUs using the following algorithm. (Algorithm 1 [46])

ALGORITHM 1 **Data generation using eCDF and Copula.**

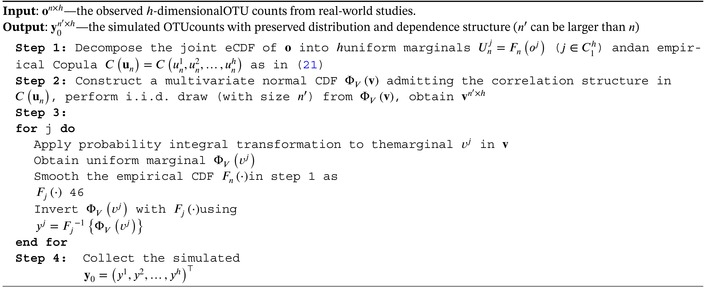

The simulated $y_{i0}$ was treated as the baseline OTUs for each subject ($i\in C_{1}^{n^{\prime }}$), where the baseline beta‐diversity $f_{i,0}=d\left(y_{i_{1}0},y_{i_{2}0}\right)$ was calculated for each pair, we centered them to create the covariate‐free “residuals” with $\varepsilon_{i}=f_{i,0}-\mu_{0},$ where the population parameter $\mu_{0}$ was obtained by the sample mean of beta‐diversity from a large sample with size 5000.

We considered three covariates, including a binary group factor $x_{i}\in \{$ D,H$\}∼Bernoulli(p_{x})$ transformed into pairwise indicators $\delta (x_{i})={\left(\delta_{\text{DD}}\left(x_{i}\right),\delta_{\text{HH}}\left(x_{i}\right),\delta_{\text{DH}}\left(x_{i}\right)\right)}^{⊤},$ a time‐invariant binary covariate $w_{i}$ $∼Bernoulli(p_{w})$ encoded and collapsed into $\delta (w_{i})={\left(\delta_{ll}\left(w_{i}\right),\delta_{12}\left(w_{i}\right)\right)}^{⊤}$ (l=1,2), and a time‐varying continuous covariate $z_{i}^{m\times 1}∼N(\mu_{z},\sum_{z})$ mapped to $g\left(z_{it}\right)$ (for each visit) using $L_{2}$ distances. We then added group‐by‐time and covariate effects, collectively denoted as $q_{it}^{⊤}\beta_{0}$, to create:

$$f_{i,t}=\exp\left(q_{it}^{⊤}\beta_{0}+\varepsilon_{i}\right)=\exp\left(q_{it}^{⊤}\beta_{0}\right)\exp\left(\varepsilon_{i}\right).$$

It is easily checked that $E\left(f_{i,t}|q_{it}\right)=\exp\left(q_{it}^{⊤}\beta_{0}\right).$

To simulate missing outcomes, we set the transition probability as a function of the time‐varying covariates $g\left(z_{it}\right)$ and constructed the weights $\pi_{i,t}(\gamma )$ as in (14) and (15), further multiplying the complete beta‐diversity outcome $f_{i,t}$ by the indicator $R_{i,t}∼Bernoulli(\pi_{i,t}(\gamma ))$ yielded the new responses whose missingness depends on observed $g\left(z_{it}\right)$. For the respective complete and missing data cases, both the response profile (M1; two visits) and continuous time (M2; three visits) Edger were evaluated (see simulation model specification details in Section S2 of Supporting Information).

#### 4.1.2 Results Under Complete and Missing Data

Implementing the multivariate UGEE for the complete and joint Weighted UGEE for missing data yielded the estimates and sandwich variances for θ during the rth MC iteration ($r\in C_{1}^{R}$), denoted as $\{{\hat{\theta }}^{\left(r\right)},$ ${\hat{\sum }}_{\theta }^{\left(r\right)}\}$. The sample mean of ${\hat{\sum }}_{\theta }^{\left(r\right)}$ served as the sample *asymptotic* variance (${\hat{\sum }}_{\theta }^{\left(asym\right)}$) while the sample variance of ${\hat{\theta }}^{\left(r\right)}$ as the *empirical* variance (${\hat{\sum }}_{\theta }^{\left(emp\right)}$), we check if they are close to ensure the sandwich variance estimator reflects the true variability. Also, we derived the Wald statistic $W_{n}^{\left(r\right)}$ for testing the null in the rth iteration and calculated the Type I error as

(22) $$\hat{\alpha }=\left(1/R\right)\overset{R}{\underset{r=1}{\sum }}I\left(W_{n}^{\left(r\right)}\geq q_{c,0.95}\right),$$

where $q_{c,0.95}$ is the 95th percentile of a central $\chi_{c}^{2}$, we then compared the coverage probability ($1-\hat{\alpha }$) with the nominal 95%.

Shown in the Table 2 are results from M1 (top panel) and M2 (lower panel) for β of main interest. All estimators from the proposed approach enjoyed the consistency and asymptotic normality indicated by the correct coverage. Here we displayed the results for a moderate sample size of 300, the same conclusions followed for smaller or larger sample sizes (100 and 600).

**TABLE 2:** Validity simulation results for response profile (M1) and continuous time (M2) Edger under the null hypotheses, both complete and missing data are considered.

	Response profile Edger (M1)							
	Complete data				MAR^c^ (missing rate: 31.8% at t=2)			
	Bias	Emp. SE^a^	Asym. SE^b^	Coverage %	Bias	Emp. SE	Asym. SE	Coverage %
$\beta_{0}$	−0.0001	0.0017	0.0017	95.4	0.0002	0.0017	0.0017	95.4
$\beta_{2}^{T}$	0.0001	0.0021	0.0021	96.2	−0.0002	0.0021	0.0021	95.2
$\beta_{12}^{G}$	0	0.0021	0.0021	95.4	−0.0001	0.0021	0.0021	95.6
$\beta_{22}^{G}$	0	0.0011	0.0010	95.4	−0.0001	0.0011	0.0011	95.2
$\beta_{2,12}^{I}$	−0.0001	0.0030	0.0028	96.4	0.0001	0.0030	0.0030	94.2
$\beta_{2,22}^{I}$	0	0.0015	0.0014	96.4	0.0001	0.0015	0.0015	95.0
$\eta_{12}$	0	0.0007	0.0007	92.8	0	0.0007	0.0007	93.6
$\xi_{1}$	0	0.0007	0.0007	94.4	0	0.0007	0.0008	94.8
	Continuous time Edger (M2)							
	Complete data				MAR (missing rate: 25.4% at t=2, 26.5% at t=3)			
	Bias	Emp. SE	Asym. SE	Coverage %	Bias	Emp. SE	Asym. SE	Coverage %
$\beta_{0}$	0.0001	0.0024	0.0022	96.7	0.0010	0.0014	0.0012	97.5
$\beta_{2}^{T}$	0	0.0006	0.0006	96.7	0.0011	0.0015	0.0013	92.6
$\beta_{12}^{G}$	0.0004	0.0053	0.0051	95.7	0.0013	0.0102	0.0103	95.4
$\beta_{22}^{G}$	0.0002	0.0030	0.0030	95.0	0.0013	0.0053	0.0056	95.6
$\beta_{2,12}^{I}$	0.0001	0.0013	0.0013	94.7	−0.0077	0.0110	0.0113	92.6
$\beta_{2,22}^{I}$	0.0001	0.0007	0.0007	95.0	−0.0081	0.0060	0.0065	94.0
$\eta_{12}$	0	0.0007	0.0007	93.7	−0.0001	0.0039	0.0038	93.6
$\xi_{1}$	0	0.0008	0.0008	94.7	0.0019	0.0034	0.0035	98.0

*Note:* All simulation results are averaged over R = 1000 iterations.

^a^ Emp. SE: empirical standard error.

^b^ Asym. SE: asymptotic standard error.

^c^ MAR: missing at random.

For response profile M1, the estimators were consistent under both complete and missing data (missing rate: 31.8% at t=2), with asymptotic variances approaching the empirical ones and the coverage close to 95%. We observed similar patterns for continuous time model M2; the slightly fluctuated coverage probabilities under the missing data (missing rate: 25.4% at t=2, 26.5% at t=3) highlighted the bias‐variance trade‐off under a parsimonious time effect, which is recommended for studies with moderate sample sizes. Both results demonstrated decent performances of the Weighted UGEE in handling missing data, even under such medium missing rates.

Lastly, our adopted data‐generating procedure retained the zero‐inflated nature of OTUs. In particular, in the real data, the percentage of zero counts is 93.93% and averaged to be 93.32% (sd = 0.004) in our simulated OTUs when simulation size $n^{\prime }=300$, further validating the propitious performance of our semiparametric inference in handling real‐world microbiome data.

### 4.2 Superiority

Although PERMANOVA and PERMDISP are not designed for longitudinal data (see Section S3 of Supporting Information for a detailed comparison), their coverage and power in visit‐specific (cross‐sectional) analyzes have been thoroughly evaluated and compared to the Edge model in prior work [20]. This work showed that semiparametric inference achieves comparable or superior power and much better computational scalability than existing methods. For instance, with an effect size of 0.352 and 100 subjects per group, PERMDISP and Edge both achieved high power (0.956 vs. 0.925). In contrast, PERMANOVA had much lower power (0.441).

Accordingly, the present study focuses on the most directly comparable longitudinal alternative, GLMM‐MiRKAT, which relates microbial composition to the host trait $x_{it}$ in a mixed effects modeling framework [6]:

(23) $$E(x_{it})=\mu_{it},\;g\left(\mu_{it}\right)=z_{it}^{⊤}\alpha +\upsilon_{i}+h(y_{it}),\;1\leq t\leq m,$$

where g(·) is a canonical link, such as logit when $x_{it}$ is binary. Here $z_{it}$ is a vector of covariates, with coefficient α representing the fixed effect, $\upsilon_{i}$ is a random intercept to account for the within‐cluster correlation, and $h(y_{it})$ is a function of microbiome effect. A matrix version by stacking all the subjects across m visits yields g(μ)=zα+υ+h(y), where g(μ) and h(y) are both (mn)‐dimensional vectors.

Since testing individual OTU effects in (23) is challenging or even impossible, transformations can be applied to h(y), yielding a vector‐valued mean zero function with variance τK, denote by $\tilde{h}(y)∼(0,\tau K),$ where $K^{\left(mn)\times (mn\right)}$ is a pairwise similarity matrix converted from the beta‐diversity and τ is an unknown constant. After controlling for covariates, the relationship between the host trait $x_{it}$ and the beta‐diversity can be tested via $H_{0}:\tau =0$ using permutations.

In the following simulation comparisons, we adopt the “dirmult” R package [47] to generate OTUs following a Dirichlet‐multinomial (DM) distribution that allows us to vary the alternative hypotheses directly. The parameters of DM, including the *taxa frequencies* π and the *scale* θ (that governs the overdispersion), were calibrated from real data containing two groups (healthy vs. diseased) from a published study [44], and we denote their parameters by $\left\{\pi_{H},\theta_{H}\right\}$ and $\left\{\pi_{D},\theta_{D}\right\}.$

Next, we varied {π,θ} to generate OTUs from different groups at differing visits, then the constructed beta‐diversity $d\left(y_{i_{1}t},y_{i_{2}t}\right):=d_{i,t}$ naturally carried the group or time effects. Different types of beta‐diversity (e.g., Bray–Curtis, Jaccard) were considered to ensure a robust model performance. Due to the unresolved computational challenge, GLMM‐MiRKAT handles missing data by casewise deletion; hence, we focused on comparing complete data.

#### 4.2.1 Results for Main Group Effect

##### 4.2.1.1 Comparable Type I Error but Semiparametric Inference Has Greater Computational Efficiency

First, we compared the Type I error and computational time of the two approaches under the null of no group effects. In Edger, $d_{i,t}$ forms the outcome, and the main group effects were tested with the null $H_{0}:$ $\beta_{\text{DD}}^{G}=\beta_{\text{DH}}^{G}=0.$ In the GLMM‐MiRKAT, the binary group index $x_{it}$ was a response, and K was Gower‐centered from the block diagonal matrix by stacking the beta‐diversity

(24) $$\left(\begin{array}{ccc} \left(d_{i,1}\right) & 0 & 0\\ 0 & \cdots & 0\\ 0 & 0 & \left(d_{i,m}\right) \end{array}\right),$$

and we tested the comparable null $H_{0}:\tau =0$ using 99, 299, 499, and 5000 permutations.

As shown in Table 3 for Bray–Curtis beta‐diversity, both methods attained reasonable Type I errors with GLMM‐MiRKAT showed occasional downward biases (e.g., 0.034) while Edger achieved reasonable performance throughout, even when the sample size was small or moderate.

**TABLE 3:** Simulation comparisons of Type I error and superiority in computational time under the null hypotheses: Edger (proposed) versus GLMM‐MiRKAT (existing).

Sample size	Type I error					Time for one iteration (in seconds)				
	Edger		GLMM‐MiRKAT			Edger	GLMM‐MiRKAT^a^			Default
	$\beta_{22}^{G}$	$\beta_{12}^{G}$	99	299	499		99	299	499	5000
40	0.052	0.047	0.044	0.049	0.049	0.068	0.154	0.328	0.519	4.933
100	0.045	0.046	0.039	0.038	0.046	0.378	0.561	1.124	1.643	13.856
200	0.044	0.043	0.034	0.045	0.043	2.102	3.161	5.058	6.977	50.285
300	0.048	0.048	0.048	0.048	0.046	5.784	9.566	13.885	17.956	110.693

^a^ GLMM‐MiRKAT is conducted with 99, 299, 499, and 5000 times of permutation.

Nonetheless, the difference in their computational time was striking (e.g., 0.068 s vs. 4.933 s for one iteration); the elapsed time of GLMM‐MiRKAT grew dramatically with the increased number of permutations, yet it is required to ensure stable results for such nonparametric tests [20]. The default permutation times in their package were hence set to 5000, resulting in 37 times the elapsed time compared with Edger, even when the sample size is only 100 (analyzes were conducted in R version 4.3.3 on an Apple M1 Pro, 34.4 GB RAM). The results for Jaccard beta‐diversity in Table S3 of the Supporting Information demonstrated a similar trend, highlighting the promise of the proposed Edger in reducing computational overhead regardless of the beta‐diversity type.

##### 4.2.1.2 Distance Regression Outperforms in Detecting Scale Difference and Covariate Adjustment

Here, we simulated OTUs with non‐zero group effects and sample sizes ranging from 40 to 300 and then computed the beta‐diversity $d_{i,t}$. Afterwards, a time‐varying normal covariate $z_{it}$ was transformed to $g\left(z_{it}\right)$ using $L_{2}$ distances, and we used $d_{i,t}\exp\left\{\xi_{0}\cdot g\left(z_{it}\right)\right\}:=f_{i,t}$ as in (8) for the new outcome involving covariate effects. The population‐level characteristics, such as the true value of $\beta_{\text{DD}}^{G}$ and $\beta_{\text{DH}}^{G}$ in Edger, were estimated by MC simulation with a large MC size of 5000.

Our comparison of main group effects centered on three cases, whose distinct patterns are shown in the histograms and the principal coordinate analysis (PCoA) plot in Figures S2–S4 of the Supporting Information:

- (Case 1) *Location* difference: The two groups were simulated with the same scale parameter θ ($\theta_{1,t}=\theta_{2,t}=\theta_{\text{HC}}$) but different taxa frequency $\pi^{\prime }$s ($\pi_{1,t}=\pi_{\text{HC}},\pi_{2,t}=\pi_{D})$.
- (Case 2) *Scale* or *dispersion* difference: The two groups had the same π ($\pi_{1,t}=\pi_{2,t}=\pi_{\text{HC}})$ but different $\theta^{\prime }$s ($\theta_{1,t}=\theta_{\text{HC}},\theta_{2,t}=\theta_{D}$).
- (Case 3) High‐dimensional covariate adjustment: we investigated their impact by replacing the previous univariate $z_{it}$ with a multivariate normal $z_{it}$ of dimension 100. The Edger regression can still directly include the $L_{2}$ distance $g\left(z_{it}\right)=$ $g\left(z_{i_{1}t},z_{i_{2}t}\right)$ as an aggregated covariate, yet this does not apply to the within‐subject GLMM‐MiRKAT. Hence, PCA was implemented first, and the top principal components (PCs) were adjusted. We focused on two scenarios:
  1. 30% of the total variance explained by top 10 PCs.
  2. 60% of the total variance explained by top 23 PCs.
  Due to the rank deficiency, GLMM‐MiRKAT did not converge when the number of explanatory variables was close to the sample size (e.g., n=40). We hence illustrated (Case 3) with only moderate to large sample sizes of 100, 200, and 300.

Table 4 indicated that in detecting (1) the location difference, Edger achieved slightly higher power when the sample size was small, and both methods yielded comparable power with larger sample sizes. However, the Edger considerably outperformed in detecting (2) the dispersion difference of the OTUs, with GLMM‐MiRKAT showing underwhelming signals; for example, the power was only 0.078 when the sample size was 40, and even when increasing it to 200, the power was still 0.29. However, Edger consistently rendered a decent power (close to 1).

**TABLE 4:** Simulation comparisons of statistical power under the alternative: Edger versus GLMM‐MiRKAT on the main group effect for beta‐diversity (Bray–Curtis).

	Power				Edger coefficient	
	GLMM‐MiRKAT			Edger	$\beta_{\text{DH}}^{G}$ (truth: −0.018)	
Sample size	99	299	499	Omnibus	Est.^a^	Std. Err.^b^
(Case 1) “*Location*” difference (main group effect)						
40	0.395	0.420	0.425	0.515	−0.018	0.021
100	0.968	0.970	0.968	0.997	−0.018	0.012
200	1	1	1	1	−0.017	0.008
250	1	1	1	1	−0.018	0.007
300	1	1	1	1	−.018	0.007

	Power				Edger coefficient	
	GLMM‐MiRKAT			Edger	$\beta_{\text{DD}}^{G}$ (truth: −0.086)	
Sample size	99	299	499	Omnibus	Est.	Std. Err.
(Case 2) “*Scale*” difference (main group effect)						
40	0.074	0.078	0.078	0.482	−0.083	0.038
100	0.120	0.130	0.130	0.920	−0.086	0.024
200	0.266	0.286	0.290	1	−0.085	0.017
250	0.386	0.422	0.450	1	−0.086	0.015
300	0.524	0.596	0.608	1	−0.086	0.014

	Power				Power			
	GLMM‐MiRKAT				GLMM‐MiRKAT			
	99	299	499	Edger	99	299	499	Edger
Sample size/total variability	(a) 30%			Omnibus	(b) 60%			Omnibus
Case 3 High‐dimensional covariate adjustment								
100	0.078	0.102	0.102	0.972	0.070	0.062	0.064	0.964
200	0.206	0.236	0.264	1	0.212	0.224	0.236	00.998
300	0.402	0.458	0.462	1	0.470	0.506	0.528	1

^a^ Est.: estimator.

^b^ Std. Err.: standard error.

The inability to adjust for sufficiently high‐dimensional covariates further deteriorated the performance of GLMM‐MiRKAT; for instance, when the sample size was 300, its power dropped from 0.608 to 0.462 when adjusting 30% of the total variability and 0.528 when adjusting 60%. Yet the Edger maintained reasonably high power (close to 1), indicating the aptitude for ensembling high‐dimensional covariates may help compensate for the smaller sample sizes.

In general, the PC adjustment in GLMM‐MiRKAT was difficult since no discernible “elbows” appeared in the scree plot to help determine the optimal cutoff, and the explained variance from the top ten PCs all ranged from 4% to 2.7% (see Figure S5 of the Supporting Information). To further investigate the information loss due to the arbitrary cutoffs when no clear rule exists, we allowed GLMM‐MiRKAT to adjust for 38 PCs to account for 80% of the total variance. However, an interesting pattern was observed: adjusting for too many PCs may undermine their performance. For example, when the sample size is 200, the power decreased from 0.236 to 0.138 when adjusted variance increased from 60% to 80%, which, can be an artifact of increased rank deficiency and unstable fit in the within‐subject‐level modeling framework.

#### 4.2.2 Results for Time‐by‐Group Interaction Effect

Next, we included time‐by‐group interaction by simulating OTUs with no baseline difference ($\pi_{1,t=1}=\pi_{2,t=1}=\pi_{\text{HC}},$ $\theta_{1,t=1}=\theta_{2,t=1}=\theta_{\text{HC}}$) but a non‐zero group effect at follow‐up visits. Cases 4 and 5 examined the respective diverging patterns:

- (Case 4) *Location* difference *over time*: The same θ but different taxa frequencies $\pi^{\prime }$s at t>1.
- (Case 5) *Scale* or *dispersion* difference *over time*: The same π but different scale parameters $\theta^{\prime }$s at t>1.

Here, we compared the null of no time‐by‐group interaction $H_{0a}:\beta_{t,\text{DD}}^{I}=\beta_{t,\text{DH}}^{I}=0$ from Edger with $H_{0b}:\tau =0$ for GLMM‐MiRKAT. Notably, both scenarios in Table 5 indicated that Edger was far more adequate when the groups diverged over time, while no baseline difference existed, which is common in many experimental studies [17]. More importantly, the superior performance of Edger is robust to the choice of beta‐diversity kernels (see Table S4 of the Supporting Information for Jaccard beta‐diversity).

**TABLE 5:** Simulation comparisons of statistical power under the alternative: Edger versus GLMM‐MiRKAT on the interaction effect for beta‐diversity (Bray–Curtis).

(Case 4) “*Location*” difference *over time*(interaction effect)						
Sample size	Power				Edger coefficient	
	GLMM‐MiRKAT			Edger	$\beta_{t,\text{DH}}^{I}$ (truth: −0.018)	
	99	299	499	Omnibus	Est.	Std. Err.
40	0.046	0.058	0.058	0.208	−0.020	0.042
100	0.034	0.034	0.042	0.698	−0.018	0.024
200	0.052	0.060	0.054	0.994	−0.019	0.017
250	0.022	0.024	0.020	1	−0.019	0.015
300	0.042	0.054	0.048	1	−0.017	0.013
(Case 5)“*Scale*” difference *over time*(interaction effect)						
	Power				Edger Coefficient	
	GLMM‐MiRKAT			Edger	$\beta_{t,\text{DD}}^{I}$ (truth: −0.086)	
Sample size	99	299	499	Omnibus	Est.	Std. Err.
40	0.034	0.048	0.044	0.168	−0.087	0.076
100	0.058	0.062	0.064	0.352	−0.087	0.048
200	0.040	0.046	0.044	0.654	−0.086	0.034
250	0.044	0.054	0.052	0.754	−0.086	0.030
300	0.030	0.040	0.044	0.858	−0.086	0.027
(Case 6) Empirical resampling: *Bootstrap* ($n_{k}=13$ vs. 22 vs. 16)						
	GLMM‐MiRKAT				Edger (Omnibus)	
IBS‐C vs. other groups	0.880				1.00	
IBS‐D vs. other groups	0.658				1.00	
(Case 7) Empirical resampling: *Stratified* ($n_{k}=20$ *per group*)						
	GLMM‐MiRKAT				Edger (Omnibus)	
IBS‐C vs. other groups	0.872				1.00	
IBS‐D vs. other groups	0.874				1.00	

#### 4.2.3 Results From Empirical Resampling

To complement those model‐based simulations, we further conducted empirical resamplings using a real‐world longitudinal microbiome dataset on irritable bowel syndrome (IBS) with five repeated visits. The original study reported subtype‐specific differences primarily based on time‐averaged beta‐diversity metrics, where the constipation‐predominant (IBS‐C) subtype showed more pronounced compositional shifts than the diarrhea‐predominant (IBS‐D) or healthy control (H) groups [48].

**FIGURE 1:**
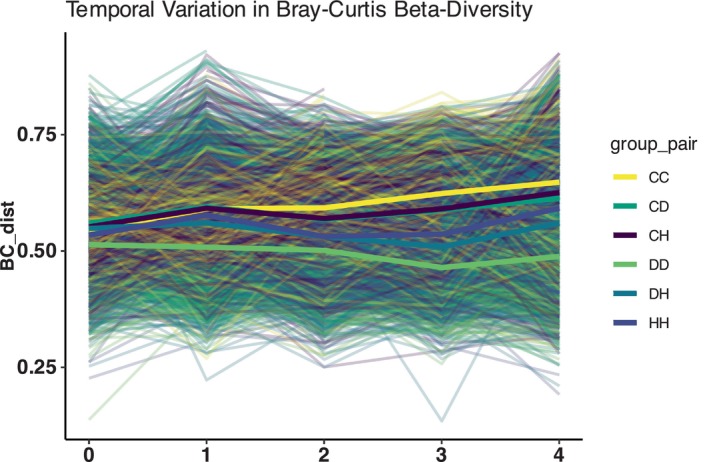
Temporal variation in Bray–Curtis beta‐diversity across IBS subtype pairs over five visits. Spaghetti plot of Bray–Curtis dissimilarity trajectories for all within‐ and between‐subtype pairs, constipation‐predominant (C), diarrhea‐predominant (D), and healthy control (H), across five repeated visits. Trajectories show pronounced within‐pair variability and temporal dynamics across all six pair types (CC, CD, CH, DD, DH, HH), underscoring the limitation of time‐averaged beta‐diversity summaries and motivating longitudinal distance‐based modeling of time‐by‐group effects.

In contrast to such time‐averaged comparisons that overlook within‐cluster temporal trends, we first applied Edger to directly model time‐by‐group effects. Our reanalyses revealed significant time main effects and longitudinal time‐by‐group interactions, with the time‐by‐location effect persisting under both complete‐case and MAR analyses (working independence, exchangeable, or unstructured, first panel of Table 6), consistent with the patterns observed in the longitudinal spaghetti plot (Figure 1). These findings indicate dynamic microbial community changes over time that extend beyond static between‐group differences.

**TABLE 6:** Real data analyses results from the proposed Edger in a longitudinal aging study and a crossover study design for the Lysine dietary intervention.

Irritable bowel syndrome (IBS) subtypes with five repeated visits				
*p*	Complete case	MAR (Independence)	MAR (Exchangeable)	MAR (Unstructured)
Time (reference: IBS‐C)	0.006	0.691	0.691	0.691
Time × group	<0.0001	0.017	0.013	0.008
Time × location	0.027	0.021	0.020	0.012
Time × scale	0.003	0.254	0.254	0.254
Aging study of human gut‐brain axis				
Parameter	Est.	Std. Err.	Wald statistics	*p*
$\beta_{0}$	−0.194	0.013	229.677	<0.0001
Time	0.014	0.018	0.589	0.443
Baseline disease vs. control pairs	0.029	0.018	2.670	0.102
Baseline mixed vs. control pairs	0.023	0.013	3.243	0.072
Time × disease vs. control pairs	−0.046	0.039	1.349	0.246
Time × mixed vs. control pairs	−0.035	0.019	3.465	0.063
Baseline alpha‐diversity	0.009	0.004	4.371	0.037
Gender	0.011	0.007	2.513	0.113
Crossover study design for dietary intervention				
Parameter	Est.	Std. Err.	Wald statistics	*p*
$\beta_{0}$	−0.691	0.043	257.510	<0.0001
Period	−0.045	0.024	3.551	0.060
Lysine vs. control pairs	−0.007	0.052	0.017	0.897
Mixed vs. control pairs	−0.016	0.025	0.400	0.527
Age	0.002	0.003	0.487	0.485
Sex	−0.019	0.019	1.006	0.316
Race	0.073	0.028	6.595	0.010

Using the complete cases from this dataset, we further conducted two empirical simulations (each with 500 replicates) to compare statistical power:

- Case 6 subject‐level bootstrap resampling, and
- Case 7 stratified resampling within the three clinical subtypes (IBS‐C, IBS‐D, H).

Both Edger and GLMM‐MiRKAT were applied to test the omnibus null of no group or time effects, controlling for age and BMI. Because GLMM‐MiRKAT only allows for binary group‐level contrasts, we align the comparisons as IBS‐C versus others and IBS‐D versus others for consistency. As shown in Table 5 (last two panels), Edger consistently achieved high empirical power (1.0), whereas GLMM‐MiRKAT exhibited moderate power (0.66–0.88).

These empirical results are consistent with model‐based simulation findings in Section 4.2.2 but under more heterogeneous real‐world conditions. In controlled simulation, GLMM‐MiRKAT lacked power when groups diverged over time without baseline differences. The current real‐data resampling illustrates that where both baseline and evolving differences coexist, GLMM‐MiRKAT recovers moderate power, yet Edger continues to demonstrate substantially higher sensitivity.

Together, these results show that Edger provides valid longitudinal inference and effectively captures time‐varying group divergence, whether driven purely by longitudinal change or by mixed temporal and baseline effects. Furthermore, unlike GLMM‐MiRKAT, which reports only a single omnibus *p* value, Edger yields interpretable coefficients (e.g., $\beta_{\text{DD}}^{I}$, $\beta_{\text{DH}}^{I}$) that delineate distinct types of differences, which may generate testable hypotheses to enhance scientific understanding. Next, we apply the proposed Edger to two real‐world studies.

## 5 Real Data Analysis

### 5.1 Aging Study of Human Gut‐Brain Axis

Our first application exemplified the dynamic impact of cognitive and mental health on the human microbiome. Driven by the increased biological heterogeneity with aging, this longitudinal study of senior housing residents (age 65+) seeks evidence for the “gut‐brain axis” via links between the human microbiome and cognitive and mental health over time [11, 18]. Participants were assessed at baseline ($n_{1}=95$) and again six months later ($n_{2}=61$). Demographic and psychosocial measures were collected at baseline, while fecal microbiome samples were obtained at both visits. Previous analyses identified significant associations between Faith's phylogenetic alpha‐diversity and measures of cognition and curiosity [49]. Building on this work, we examined whether baseline alpha‐diversity influences longitudinal patterns of Jaccard beta‐diversity, and whether these dynamics differ by cognitive and curiosity profiles. Specifically, individuals with mild cognitive impairment (MCI) and high curiosity were identified using baseline MoCA (montreal cognitive assessment) scores [50] and CEI‐II (curiosity and exploration inventory‐II) measures [51], applying established clinical cutoffs. This subgroup indicator served as the primary grouping variable.

We specified the Edger model to include baseline differences in alpha‐diversity (quantified using the $L_{2}$ distance), a group‐by‐time interaction, and gender as a covariate. The missingness mechanism was modeled as dependent on alpha‐diversity. For illustrative purposes, results were not adjusted for multiple comparisons.

Although no significant group‐by‐time interaction was detected (overall p=0.119), we observed a significant positive association between baseline differences in alpha‐diversity and longitudinal trajectories of beta‐diversity (β=0.009, p=0.037; middle panel of Table 6). Biologically, this finding suggests that individuals entering the study with more distinct within‐subject diversity profiles tended to exhibit sustained or increasing between‐subject dissimilarity over time, consistent with progressive divergence in community‐level organization.

At baseline, mixed pairs showed modestly higher beta‐diversity than HH pairs (β=0.023, p=0.072), indicating greater initial heterogeneity between groups. This difference attenuated over time (β=−0.035, p=0.063), suggesting partial convergence or stabilization of microbial community structure during follow‐up. Although marginally significant, this pattern is consistent with a baseline location shift followed by temporal convergence.

**FIGURE 2:**
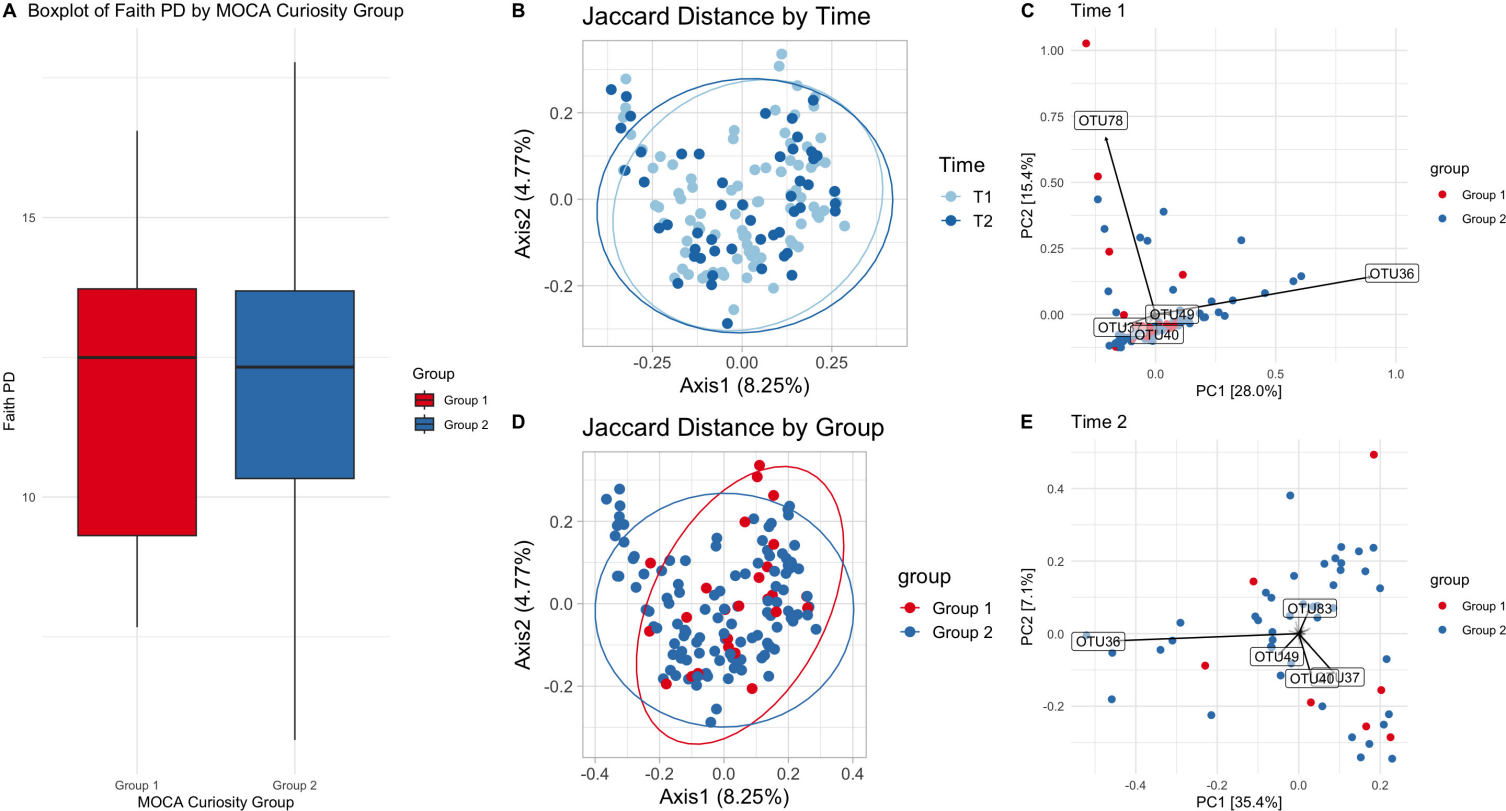
Microbiome alpha‐ and beta‐diversity patterns by MOCA curiosity group in a longitudinal aging cohort. (A) Boxplots of Faith's phylogenetic diversity (alpha‐diversity) at baseline by MOCA curiosity group (Group 1: MCI/high curiosity; Group 2: healthy controls), showing comparable medians but greater spread in Group 1. (B, D) Principal coordinate analysis (PCoA) of Jaccard beta‐diversity across all observations, plotted separately by time point (B: T1 vs. T2 overlaid) and by group (D: Group 1 vs. Group 2), with 95% concentration ellipses. Groups show partially overlapping but directionally distinct centroids, consistent with a modest baseline location shift followed by partial convergence over six months. (C, E) PCA biplots at Time 1 and Time 2, respectively, with loadings for key OTUs annotated to highlight the taxa most contributing to between‐group compositional differences at each visit.

These results are supported by the PCoA shown in Figure 2, which reveals subtle but coherent group separation in location. Taken together, the findings suggest that microbiome differences associated with cognitive status and curiosity are most evident at study entry and may diminish with aging.

### 5.2 Crossover Study Design for Dietary Intervention

The second application illustrates the use of Edger in a two‐period, two‐sequence crossover design with shotgun metagenomic microbiome data. Motivated by evidence that gut microbial communities can respond rapidly to dietary and nutrient perturbations, this study randomized 45 participants to receive either a Lysine supplement or a placebo (more study details are included in Section S4 of the Supporting Information). The Edger framework was applied to evaluate *carryover*, *intervention*, and *period* effects on beta‐diversity within this crossover setting [30].

We first fit a model including the period‐by‐group interaction, adjusting for sex, race, and age. The interaction term was not statistically significant (Wald = 0.512, p = 0.480), providing no evidence of carryover effects. Accordingly, we proceeded with a reduced model to assess the main intervention and period effects.

**FIGURE 3:**
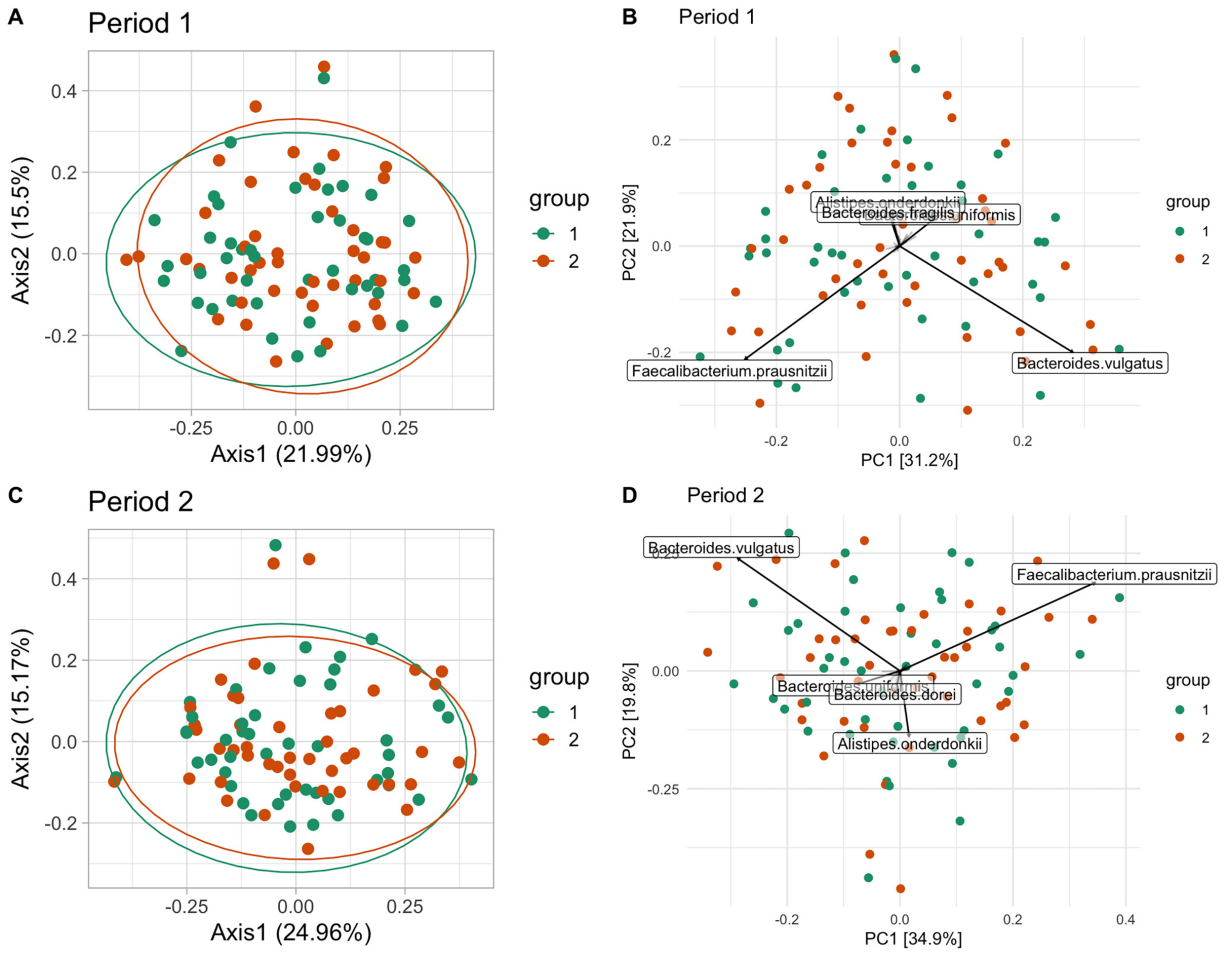
Bray–Curtis Beta‐Diversity by Treatment Group Across Periods in a Lysine Supplementation Crossover Trial. (A, C) PCoA plots of Bray–Curtis dissimilarity at Period 1 and Period 2, respectively, with 95% concentration ellipses by group (Group 1: Lysine; Group 2: placebo). Centroids are near‐overlapping at both periods, consistent with the absence of a significant intervention effect or carryover. Ellipses appear slightly more compact in Period 2, reflecting the marginally significant reduction in beta‐diversity over time independent of treatment. (B, D) Corresponding PCA biplots annotating the taxa most influential in driving compositional variation at each period. Shifts in the relative loadings of these taxa across periods suggest period‐dependent reorganization of community structure despite stable group‐level centroids.

Visual inspection of the Bray–Curtis PCoA (Figure 3) revealed no apparent separation between intervention and control groups at either period, with near‐overlapping group centroids. Consistent with this observation, Edger results (Table 6, last panel) indicated that although participants receiving Lysine exhibited slightly greater within‐group similarity than placebo pairs, this effect was not statistically significant (β=−0.007, p=0.897), suggesting a weak trend toward microbial community convergence under Lysine supplementation. The mixed‐pair contrast was also negative (β=−0.016, p=0.527), indicating marginally greater similarity between Lysine‐ and placebo‐treated participants than among placebo pairs alone. Together, these patterns are consistent with partial convergence or shared stabilization of gut microbial communities [27, 28], although the modest sample size limits statistical power.

Interestingly, the period effect was marginally significant, with reduced beta‐diversity observed in the second period (β=−0.045, p = 0.060), suggesting increased microbial similarity over time independent of treatment assignment. Similar trends were observed for alternative beta‐diversity measures (e.g., Jaccard; results not shown). The directions of these effects are consistent with prior experimental evidence, including animal studies reporting reduced microbial diversity following Lysine supplementation [52].

Overall, this application demonstrates that Edger can be readily adapted to crossover designs, enabling simultaneous assessment of intervention, period, carryover, and covariate effects within a unified modeling framework. Notably, race emerged as a significant covariate (β = 0.073, p = 0.010), reflecting residual imbalance despite randomization. This finding underscores the importance of covariate adjustment in randomized microbiome studies and highlights how Edger can inform covariate selection and study design in future intervention trials.

## 6 Discussion

The rapid growth of longitudinal studies in human microbiome research calls for analytical tools to accommodate repeated measures and missing data while providing robust inferential insights. The proposed Edger, a semiparametric distance regression for repeated outcomes, promptly addresses these challenges and demonstrates strong empirical performance.

By focusing on beta‐diversity, a key microbial community index that captures between‐subject variability, Edger provides complementary insights into treatment‐related resilience and community convergence or divergence [53, 54], particularly in longitudinal microbiome studies where community‐level dynamics are of primary interest. By aggregating individual taxa and phylogenetic information into ecological distances, Edger enables researchers to examine dynamic microbiome patterns and microbiome‐host interactions more effectively, while offering valuable biological insights regarding the convergence, divergence, and stability of the community composition.

This aggregating feature is particularly advantageous in microbiome studies involving numerous covariates but with limited sample sizes. In studies where covariates from other ‐omic sources or electronic health records (EHR) are incorporated [55], conventional approaches often apply dimension reduction techniques to allow for within‐subject modeling, potentially leading to information loss and irreproducible results [36, 37, 56]. In contrast, Edger provides a between‐subject alternative that aggregates covariates while maintaining statistical power, as demonstrated in our simulations.

Further, Edger extends the generalized estimating equations (GEE) framework to repeated distance outcomes, disentangling within‐ and between‐pair correlations while allowing for flexible modeling of time‐dependent structures. Using U‐statistics, the proposed U‐statistics‐based GEE effectively addresses the inferential challenges arising from non‐independent pairwise outcomes, achieving higher statistical power and improved computational efficiency. Notably, in scenarios where groups exhibit no baseline differences but diverge over time, Edger consistently demonstrates promising power performance.

Another key strength of Edger lies in its ability to handle missing data. Unlike methods restricted to complete‐case analysis, which can bias inference, Edger leverages all available information under the more realistic missing at random (MAR) assumption to mitigate selection bias [17]. The main outcome model flexibly accommodates both discrete and continuous time through quadratic, spline‐based, or change‐point functions. To simplify implementation, a monotone missing‐data structure is imposed on the nuisance model, following standard longitudinal practice [16, 21, 24]. However, the Weighted UGEE estimator will remain valid under more general missing patterns (e.g., intermittent missing) and can be extended via weighting adjustments [21]. Moreover, our joint inference technique integrates missing data models within the primary modeling framework, eliminating the need for conventional variance adjustments [24]. The algorithm has been optimized using Rcpp and is available for implementation.

Beyond practical considerations, we emphasize that rigorous inferential conclusions should be prioritized over data‐driven selection of certain optimal distance metrics. We emphasize the importance of structuring hypothesis tests based on domain knowledge rather than empirical tuning, which may help mitigate the “*p* value‐hacking” concerns and improve the reproducibility of microbiome studies [7, 56].

While our described model primarily focuses on distance measures derived from microbial abundances (i.e., OTUs), Edger is readily generalizable to functional microbiome data, where distances can be constructed from microbial gene families using metatranscriptomic abundances [57]. Applying Edger to such outcomes could provide deeper insights into the functional pathways of microbial communities. Additionally, our framework facilitates the integration of multi‐omics data, such as genomic, metabolomic, and proteomic layers [39]. Beyond microbiome studies, Edger can also extend to multi‐modality data, including other modalities such as neuroimaging and wearable device signals [10, 58]. In particular, Edger expands the scope of cross‐sectional similarity‐based multimodal regression [38] to accommodate repeated high‐dimensional observations, opening new avenues for studying dynamic interactions across multiple biological and physiological domains.

Given the complex structure of OTUs, future extensions could explicitly address potential sparsity‐induced zero inflation at the distance level. For example, through zero‐adjusted beta‐diversity metrics [59, 60] or mixture‐based working variance structures [61, 62], to better capture heterogeneity across sample pairs. Another promising direction is to enhance the robustness of the Weighted UGEE via semiparametric efficiency augmentation, which enables doubly robust estimation under potential misspecification of nuisance models [21, 63]. Although MAR is a widely accepted assumption, future work should also explore sensitivity analyses for missing not at random (MNAR) scenarios in real‐world applications.

Finally, this work represents an early‐phase methodological investigation [64], aimed at demonstrating feasibility and illustrating the potential of the proposed framework under realistic data‐generating conditions. While simulation and real‐data analyses indicate strong robustness and interpretability, broader evaluations across larger and more diverse datasets will be valuable for establishing generalizability and comparative performance.

## Funding

The authors have nothing to report.

## Conflicts of Interest

The authors declare no conflicts of interest.

## Supporting information




**Section S1:** Theorem 1.
**Section S2:** Simulation details.
**Table S1:** Comparison of overall (omnibus) group difference on IBS study.
**Section S3:** Comparison scross PERMDISP, PERMANOVA, GLMM‐MIRKAT, and the proposed approach.
**Section S4:** Details of the dietary intervention study.
**Figure S1:** Examples demonstrating “location” (left) and “dispersion” (right) group differences using principal coordinates analysis (PCoA).
**Table S2:** Simulation results for response profile (M1) and continuous time (M2) Edger under the null hypotheses using Jaccard beta‐diversity.
**Figure S2:** The histograms of microbiome OTUs from the real study data that contains two groups: HC vs. diseased (AUD.AH).
**Figure S3:** The histograms (a) and PCoA plot (b) of simulated OTUs for the two groups at t=1 in Case 1 to demonstrate their differences in location, or the centers.
**Figure S4:** The histograms (a) and PCoA plot (b) of simulated OTUs for the two groups at t=1 in Case 2 to demonstrate their differences in variability, or dispersion.
**Figure S5:** The scree plot of top 10 PCs from the simulated 100 covariates in Case 3 of high‐dimensional covariate adjustment, showing no definitive cut point from the PCA.
**Table S3:** Simulation comparisons of statistical power under the alternative: Edger (proposed) versus GLMM‐MiRKAT (existing) using Jaccard beta‐diversity.
**Table S4:** Empirical resampling study to evaluate Type I error under the null hypothesis using permutation of group labels of IBS study.
Proof of Theorem 1, simulation details, additional Figures and Tables referenced in the Main Manuscript are available in the Supporting Information. Edger is implemented in R and optimized using Rcpp, which are available at GitHub. The repository includes R and Rcpp scripts, example datasets, and reproducible instructions for all results presented in this manuscript.

## Data Availability

The data that support the findings of this study are openly available in National Center for Biotechnology Information at https://www.ncbi.nlm.nih.gov/bioproject/, reference number PRJNA517994.
