# Supplementary material for: Novel Distance Regression for Repeated Outcomes With Missing Data: Applications to Longitudinal and Crossover Studies of Microbiome Beta‐Diversity
Source: Stat Med. 2026 Jul 2;45(15-17):e70654. doi: 10.1002/sim.70654 (PMC13328406; doi:10.1002/sim.70654)
Supplement: Supplementary file 1 — Section S1: Theorem 1. Section S2: Simulation details. Table S1: Comparison of overall (omnibus) group difference on IBS study. Section S3: Comparison scross PERMDISP, PERMANOVA, GLMM‐MIRKAT, and the proposed approach. Section S4: Details of the dietary intervention study. Figure S1: Examples demonstrating “location” (left) and “dispersion” (right) group differences using principal coordinates analysis (PCoA). Table S2: Simulation results for response profile (M1) and continuous time (M2) Edger under the null hypotheses using Jaccard beta‐diversity. Figure S2: The histograms of microbiome OTUs from the real study data that contains two groups: HC vs. diseased (AUD.AH). Figure S3: The histograms (a) and PCoA plot (b) of simulated OTUs for the two groups at t=1 in Case 1 to demonstrate their differences in location, or the centers. Figure S4: The histograms (a) and PCoA plot (b) of simulated OTUs for the two groups at t=1 in Case 2 to demonstrate their differences in variability, or dispersion. Figure S5: The scree plot of top 10 PCs from the simulated 100 covariates in Case 3 of high‐dimensional covariate adjustment, showing no definitive cut point from the PCA. Table S3: Simulation comparisons of statistical power under the alternative: Edger (proposed) versus GLMM‐MiRKAT (existing) using Jaccard beta‐diversity. Table S4: Empirical resampling study to evaluate Type I error under the null hypothesis using permutation of group labels of IBS study. Proof of Theorem 1, simulation details, additional Figures and Tables referenced in the Main Manuscript are available in the Supporting Information. Edger is implemented in R and optimized using Rcpp, which are available at GitHub. The repository includes R and Rcpp scripts, example datasets, and reproducible instructions for all results presented in this manuscript. [file SIM-45-0-s001.pdf]

Received: Added at production | Revised: Added at production | Accepted: Added at production

DOI: xxx/xxxx

ARTICLE TYPE

Supplementary Materials for Novel Distance Regression for Repeated Outcomes with Missing Data: Applications to Longitudinal and Crossover Studies of Microbiome Beta-Diversity

Jinyuan Liu[1] | Ke Xu[1] | Jane F. Ferguson[2] | Kaidi Kang[1] | Yue Wang[3] | Yuqi Qiu[4] | Lucy Shao[5] | Shengjia Tu[5] | Tanya T. Nguyen[6,7,8] | Tuo Lin[9] | Xinlian Zhang[5]

[1]Department of Biostatistics, Vanderbilt University, 2525 West End Ave, TN 37203, Tennessee, U.S.A.

[2]Division of Cardiovascular Medicine, Department of Medicine, Vanderbilt University, 2525 West End Ave, TN 37203, Tennessee, U.S.A.

[3]Department of Biostatistics and Informatics, University of Colorado Anschutz Medican Campus, 13001 E 17th Pl, CO 80045, Colorado, U.S.A.

[4]KLATASDS-MOE, School of Statistics, East China Normal University, 3363 Zhongshan Rd (N), 200050, Shanghai, China

[5]Division of Biostatistics and Bioinformatics, UC San Diego, 9500 Gilman Dr, CA 92093, California, U.S.A.

[6]Center for Microbiome Innovation, UC San Diego, 9500 Gilman Dr, CA 92093, California, U.S.A.

[7]Department of Psychiatry, UC San Diego, 9500 Gilman Dr, CA 92093, California, U.S.A.

[8]Stein Institute for Research on Aging, UC San Diego, 9500 Gilman Dr, CA 92093, California, U.S.A.

[9]Department of Biostatistics, University of Florida, 2004 Mowry Road, FL 32611, Florida, U.S.A.

Correspondence
Jinyuan Liu, Department of Biostatistics, Vanderbilt University
Email: jinyuan.liu@vumc.org

Present address
2525 West End Ave, TN 37203, Tennessee, U.S.A.

Abstract

The human microbiome plays a crucial role in health, but understanding its dynamic relationship with the host requires regular monitoring. Beyond challenges such as high dimensionality and sparsity, additional complexities arise, particularly within-cluster correlation from repeated measures and pervasive missing data. To address these issues, we develop Edger, a novel distance regression method for modeling community-level beta-diversity dynamics and their interactions with treatment or host physiology. By focusing on beta-diversity, a distance metric between microbial profiles, Edger (*E*nsembled semiparametric *d*istance-based *g*eneralized *e*stimation for *r*epeated outcomes) directly models these distances as repeated outcomes, yielding interpretable coefficients and enabling a covariate batching strategy to mitigate omitted variable bias. Our semiparametric inference framework eliminates the need for time-consuming permutation tests, distinguishes between-cluster heterogeneity from within-cluster fluctuations, and allows flexible specification of working correlation structures. To handle missing data, we assume a missing-at-random (MAR) mechanism and incorporate a between-subject propensity score in the repeated distance regression to provide seamless joint inference, ensuring robust variance estimation without casewise deletion. Additionally, we introduce an algorithm to generate synthetic data from real-world microbial counts while preserving their zero-inflated and correlated nature. Edger demonstrates superior inferential power and computational efficiency through our numerical studies and real-world applications, making it a valuable tool for uncovering microbiome-host interactions and advancing multi-omics data integration.

KEYWORDS
Between-subject outcome, Feature aggregation, Missing at random, U-statistics, Semiparametric inference, Weighted estimating equation

 © 2023 Copyright Holder Name

1 | THEOREM 1

1.1 | Proof

We first prove the consistency, which amounts to show $E\{\mathbf{W}_n(\boldsymbol{\theta})\} = \mathbf{0}$ where

$$\mathbf{W}_n(\boldsymbol{\theta}) = \sum_{\mathbf{i}\in C_2^n} \mathbf{W}_{n,\mathbf{i}}(\boldsymbol{\theta}) = \sum_{\mathbf{i}\in C_2^n} \mathbf{G}_\mathbf{i}\mathbf{V}_\mathbf{i}^{-1}\boldsymbol{\Delta}_\mathbf{i}(\gamma)\mathbf{S}_\mathbf{i}(\boldsymbol{\theta}) = \mathbf{0}.$$

By the tower law, or the law of the iterated conditional expectations,

$$E\{\mathbf{W}_n(\boldsymbol{\theta})\} = E(\mathbf{G}_\mathbf{i}\mathbf{V}_\mathbf{i}^{-1}E(\boldsymbol{\Delta}_\mathbf{i}(\gamma)\mathbf{S}_\mathbf{i}(\boldsymbol{\theta})) = E\left[\mathbf{G}_\mathbf{i}\mathbf{V}_\mathbf{i}^{-1}E\{\boldsymbol{\Delta}_\mathbf{i}(\gamma)\mathbf{S}_\mathbf{i}(\boldsymbol{\theta}) \mid \mathbf{q}_\mathbf{i}\}\right],$$

first write $\mathbf{S}_\mathbf{i} = (\mathbf{S}_{\mathbf{i},11}^\top, \mathbf{S}_{\mathbf{i},22}^\top)^\top$ with $\mathbf{S}_{\mathbf{i},11} = \left((f_{\mathbf{i},1} - h_{\mathbf{i},1}), ..., (f_{\mathbf{i},m} - h_{\mathbf{i},m})\right)^\top$ the residual in the main response module and $\mathbf{S}_{\mathbf{i},22} = \left(\left(R_{\mathbf{i},2} - p_{\mathbf{i},2}\right), ..., \left(R_{\mathbf{i},m} - p_{\mathbf{i},m}\right)\right)^\top$ for the missing data module. Further,

$$\boldsymbol{\Delta}_{\mathbf{i},11}(\gamma)\mathbf{S}_{\mathbf{i},11}(\boldsymbol{\theta}) = \left(\frac{R_{\mathbf{i},1}}{\pi_{\mathbf{i},1}(\gamma)}(f_{\mathbf{i},1} - h_{\mathbf{i},1}), ..., \frac{R_{\mathbf{i},m}}{\pi_{\mathbf{i},m}(\gamma)}(f_{\mathbf{i},m} - h_{\mathbf{i},m})\right)^\top,$$
$$\boldsymbol{\Delta}_{\mathbf{i},22}\mathbf{S}_{\mathbf{i},22}(\boldsymbol{\theta}) = \left(\left(R_{\mathbf{i},2} - p_{\mathbf{i},2}\right), ..., \left(R_{\mathbf{i},m} - p_{\mathbf{i},m}\right)\right)^\top.$$

In the main response module, we evaluate $E\left\{\boldsymbol{\Delta}_{\mathbf{i},11}(\gamma)\mathbf{S}_{\mathbf{i},11}(\boldsymbol{\theta}) \mid \mathbf{q}_\mathbf{i}\right\}$ elementwisely, where for each $1 \leq t \leq m$

$$\begin{aligned}
E\left\{\frac{r_{\mathbf{i},t}}{\pi_{\mathbf{i},t}(\gamma)}(f_{\mathbf{i},t} - h_{\mathbf{i},t}) \mid \mathbf{q}_\mathbf{i}\right\} &= E\left[E\left\{\frac{r_{\mathbf{i},t}}{\pi_{\mathbf{i},t}(\gamma)}(f_{\mathbf{i},t} - h_{\mathbf{i},t}) \mid \mathbf{q}_\mathbf{i}\right\} \mid \mathbf{q}_\mathbf{i}, \mathbf{f}_\mathbf{i}\right] \\
&= E\left[E\left\{\frac{r_{\mathbf{i},t}}{\pi_{\mathbf{i},t}(\gamma)}(f_{\mathbf{i},t} - h_{\mathbf{i},t}) \mid \mathbf{q}_\mathbf{i}, \mathbf{f}_\mathbf{i}\right\} \mid \mathbf{q}_\mathbf{i}\right] \\
&= E\left[(f_{\mathbf{i},t} - h_{\mathbf{i},t})\frac{1}{\pi_{\mathbf{i},t}(\gamma)}E\left(r_{\mathbf{i},t} \mid \mathbf{q}_\mathbf{i}, \mathbf{f}_\mathbf{i}\right) \mid \mathbf{q}_\mathbf{i}\right] \\
&= E\left[(f_{\mathbf{i},t} - h_{\mathbf{i},t})\frac{1}{\pi_{\mathbf{i},t}(\gamma)}E\left(r_{\mathbf{i},t} \mid \mathbf{q}_{\mathbf{i},t^-}, \mathbf{f}_{\mathbf{i},t^-}\right) \mid \mathbf{q}_\mathbf{i}\right] \text{ by MAR} \\
&= E\left[(f_{\mathbf{i},t} - h_{\mathbf{i},t})\frac{1}{\pi_{\mathbf{i},t}(\gamma)}\pi_{\mathbf{i},t}(\gamma) \mid \mathbf{q}_\mathbf{i}\right] \\
&= E\left[\left(f_{\mathbf{i},t} - h_{\mathbf{i},t}\right) \mid \mathbf{q}_\mathbf{i}\right] = 0
\end{aligned}$$

In the missing data module, for each $2 \leq s \leq m$, $p_{\mathbf{i},s} = \Pr\left(R_{\mathbf{i},s} = 1 \mid R_{\mathbf{i},(s-1)} = 1, \mathbf{q}_{\mathbf{i},s^-}, \mathbf{f}_{\mathbf{i},s^-}\right)$, hence,

$$\begin{aligned}
E\left(\left(r_{\mathbf{i},s} - p_{\mathbf{i},s}\right) \mid \mathbf{q}_\mathbf{i}\right) &= E\left[E\left(\left(r_{\mathbf{i},s} - p_{\mathbf{i},s}\right) \mid \mathbf{q}_\mathbf{i}\right) \mid r_{\mathbf{i},(t-1)} = 1, \mathbf{q}_{\mathbf{i},t^-}, \mathbf{f}_{\mathbf{i},t^-}\right] \\
&= E\left[\left(E\left(r_{\mathbf{i},s} \mid r_{\mathbf{i},(t-1)} = 1, \mathbf{q}_{\mathbf{i},t^-}, \mathbf{f}_{\mathbf{i},t^-}\right) - p_{\mathbf{i},s}\right) \mid \mathbf{q}_\mathbf{i}\right] \\
&= E\left[\left(p_{\mathbf{i},s} - p_{\mathbf{i},s}\right) \mid \mathbf{q}_\mathbf{i}\right] = 0
\end{aligned}$$

We then prove asymptotic normality. Without loss of generality, consider the normalized quantity $\binom{n}{2}^{-1}\mathbf{W}_n$ and continue to denote it as $\mathbf{W}_n$. We have:

$$\sqrt{n}\left(\widehat{\boldsymbol{\theta}} - \boldsymbol{\theta}\right) = \left(-\frac{\partial}{\partial\boldsymbol{\theta}}\mathbf{W}_n\right)^{-\top}\{\sqrt{n}\mathbf{W}_n - \left(\frac{\partial}{\partial\boldsymbol{\varphi}}\mathbf{W}_n\right)^\top\sqrt{n}\left(\widehat{\boldsymbol{\varphi}} - \boldsymbol{\varphi}\right)\} + \mathbf{o}_p(1),$$

where by definition,

$$\frac{\partial}{\partial\boldsymbol{\theta}}\mathbf{W}_n = \mathbf{B}^\top + \mathbf{o}_p(1), \quad \frac{\partial}{\partial\boldsymbol{\varphi}}\mathbf{W}_n = \mathbf{o}_p(1).$$

It follows from properties of multivariate U-statistics that:

$$\sqrt{n}\mathbf{W}_n = \sqrt{n}\frac{2}{n}\sum_{i_1=1}^{n} E\left(\mathbf{W}_{n,\mathbf{i}} \mid \mathbf{y}_{i_1}, \mathbf{q}_{i_1}\right) + \mathbf{o}_p(1) = \sqrt{n}\frac{2}{n}\sum_{i_1=1}^{n} \mathbf{v}_{i_1} + \mathbf{o}_p(1).$$

It then follows that

$$\sqrt{n}\left(\widehat{\boldsymbol{\theta}} - \boldsymbol{\theta}\right) = -\mathbf{B}^{-\top}\frac{\sqrt{n}}{n}\sum_{i_1=1}^{n}\left(2\mathbf{v}_{i_1}\right) + \mathbf{o}_p(1) \to_d N(\mathbf{0}, \boldsymbol{\Sigma}_{\boldsymbol{\theta}}).$$

Further, define

$$\widetilde{\mathbf{G}}_{\mathbf{i},11} = \frac{\partial}{\partial\boldsymbol{\beta}}\mathbf{h}_{\mathbf{i}}(\boldsymbol{\beta}), \ \ \widetilde{\mathbf{G}}_{\mathbf{i},22} = \frac{\partial}{\partial\boldsymbol{\gamma}}\mathbf{p}_{\mathbf{i}}(\boldsymbol{\gamma}),$$

it is shown that

$$\frac{\partial}{\partial\boldsymbol{\theta}}\mathbf{W}_{n,\mathbf{i}} = \frac{\partial}{\partial\boldsymbol{\theta}}\begin{pmatrix}\mathbf{W}_{n,\mathbf{i},11}(\boldsymbol{\beta},\boldsymbol{\gamma}) \\ \mathbf{W}_{n,\mathbf{i},22}(\boldsymbol{\gamma})\end{pmatrix} = \begin{pmatrix}\frac{\partial}{\partial\boldsymbol{\beta}}\mathbf{W}_{n,\mathbf{i},11}(\boldsymbol{\beta},\boldsymbol{\gamma}) & \frac{\partial}{\partial\boldsymbol{\gamma}}\mathbf{W}_{n,\mathbf{i},11}(\boldsymbol{\beta},\boldsymbol{\gamma}) \\ \frac{\partial}{\partial\boldsymbol{\beta}}\mathbf{W}_{n,\mathbf{i},22}(\boldsymbol{\gamma}) & \frac{\partial}{\partial\boldsymbol{\gamma}}\mathbf{W}_{n,\mathbf{i},22}(\boldsymbol{\gamma})\end{pmatrix},$$

further simplified to be

$$\begin{pmatrix}-\widetilde{\mathbf{G}}_{\mathbf{i},11}\boldsymbol{\Delta}_{\mathbf{i},11}(\boldsymbol{\gamma})V_{\mathbf{i},11}^{-1}(\boldsymbol{\beta})\widetilde{\mathbf{G}}_{\mathbf{i},11}^{\top} & \widetilde{\mathbf{G}}_{\mathbf{i},11}V_{\mathbf{i},11}^{-1}(\boldsymbol{\beta})S_{\mathbf{i},11}(\boldsymbol{\beta})\left[\frac{\partial}{\partial\boldsymbol{\gamma}}\boldsymbol{\Delta}_{\mathbf{i},11}(\boldsymbol{\gamma})\right] \\ \mathbf{0} & -\widetilde{\mathbf{G}}_{\mathbf{i},22}\boldsymbol{\Delta}_{\mathbf{i},22}V_{\mathbf{i},22}^{-1}(\boldsymbol{\gamma})\widetilde{\mathbf{G}}_{\mathbf{i},22}^{\top}\end{pmatrix},$$

and hence, $\mathbf{B} = E\left(\frac{\partial}{\partial\boldsymbol{\theta}}\mathbf{W}_{n,\mathbf{i}}\right)$ yields a form of

$$\begin{pmatrix}\mathbf{B}_{11} & \mathbf{B}_{12} \\ \mathbf{0} & \mathbf{B}_{22}\end{pmatrix},$$

and

$$\mathbf{B}^{-1} = \begin{pmatrix}\mathbf{B}_{11} & \mathbf{B}_{12} \\ \mathbf{0} & \mathbf{B}_{22}\end{pmatrix}^{-1} = \begin{pmatrix}\mathbf{B}_{11}^{-1} & -\mathbf{B}_{11}^{-1}\mathbf{B}_{12}\mathbf{B}_{22} \\ \mathbf{0} & \mathbf{B}_{22}^{-1}\end{pmatrix},$$

to drive the partition

$$\boldsymbol{\Sigma}_{\boldsymbol{\theta}} = \mathbf{B}^{-1}\boldsymbol{\Sigma}_W\mathbf{B}^{-1} = \begin{pmatrix}\mathbf{B}_{11}^{-1}(\boldsymbol{\Sigma}_{\beta}^{W} - \mathbf{B}_{12}\mathbf{B}_{22}^{-1}\boldsymbol{\Sigma}_{\gamma\beta}^{W})\mathbf{B}_{11}^{-1} & \mathbf{B}_{11}^{-1}\boldsymbol{\Sigma}_{\beta\gamma}^{W}\mathbf{B}_{22}^{-1} \\ \mathbf{B}_{22}^{-1}\boldsymbol{\Sigma}_{\gamma\beta}^{W}\mathbf{B}_{11}^{-1} & \mathbf{B}_{22}^{-1}(\boldsymbol{\Sigma}_{\gamma}^{W} - \boldsymbol{\Sigma}_{\gamma}^{W}\mathbf{B}_{11}^{-1}\mathbf{B}_{12})\mathbf{B}_{22}^{-1}\end{pmatrix} = \begin{pmatrix}\boldsymbol{\Sigma}_{\beta} & \boldsymbol{\Sigma}_{\beta\gamma} \\ \boldsymbol{\Sigma}_{\gamma\beta} & \boldsymbol{\Sigma}_{\gamma}\end{pmatrix}.$$

1.2 | Estimation of the sandwich variance estimator

In practice, the covariance matrix is consistently estimated using the empirical sandwich estimator

$$\widehat{\boldsymbol{\Sigma}}_{\boldsymbol{\theta}} = \widehat{\mathbf{B}}^{-1}\left(\frac{4}{n}\sum_{i_1=1}^{n}\widehat{\mathbf{v}}_{i_1}\widehat{\mathbf{v}}_{i_1}^{\top}\right)\widehat{\mathbf{B}}^{-1},$$

where $\widehat{\mathbf{v}}_{i_1}$ denotes the empirical analog of $E\left(\mathbf{W}_{n,\mathbf{i}} \mid \mathbf{y}_{i_1}, \mathbf{q}_{i_1}\right)$ evaluated at the estimated parameters $\widehat{\boldsymbol{\theta}}$.

Partitioning $\widehat{\boldsymbol{\Sigma}}_{\boldsymbol{\theta}}$ conformably with $\boldsymbol{\theta} = \left(\boldsymbol{\beta}^{\top}, \boldsymbol{\gamma}^{\top}\right)^{\top}$,

$$\widehat{\boldsymbol{\Sigma}}_{\boldsymbol{\theta}} = \begin{pmatrix}\widehat{\boldsymbol{\Sigma}}_{\beta} & \widehat{\boldsymbol{\Sigma}}_{\beta\gamma} \\ \widehat{\boldsymbol{\Sigma}}_{\gamma\beta} & \widehat{\boldsymbol{\Sigma}}_{\gamma}\end{pmatrix},$$

the upper-left block $\widehat{\boldsymbol{\Sigma}}_{\beta}$ is the covariance for the regression parameters of primary interest.

Further, partitioning $\boldsymbol{\Sigma}_W$ as

$$\boldsymbol{\Sigma}_W = \begin{pmatrix}\boldsymbol{\Sigma}_{\beta}^{W} & \boldsymbol{\Sigma}_{\beta\gamma}^{W} \\ \boldsymbol{\Sigma}_{\gamma\beta}^{W} & \boldsymbol{\Sigma}_{\gamma}^{W}\end{pmatrix},$$

then

$$\boldsymbol{\Sigma}_{\beta} = \mathbf{B}_{11}^{-1}(\boldsymbol{\Sigma}_{\beta}^{W} - \mathbf{B}_{12}\mathbf{B}_{22}^{-1}\boldsymbol{\Sigma}_{\gamma\beta}^{W})\mathbf{B}_{11}^{-1},$$

which provides the exact form of asymptotic covariance for the regression coefficients.

2 | SIMULATION DETAILS

Without loss of generality, consider a binary group factor $x_i \in \{1, 2\}$, which results in a three-level pairwise indicator for the $\mathbf{i}$th pair: $\boldsymbol{\delta}(\mathbf{x}_{\mathbf{i}t}) = \left(\delta_{11}(\mathbf{x}_{\mathbf{i}t}), \delta_{22}(\mathbf{x}_{\mathbf{i}t}), \delta_{12}(\mathbf{x}_{\mathbf{i}t})\right)^\top$. We included two additional covariates with one time-invariant binary w_i encoded and collapsed into $\boldsymbol{\delta}(\mathbf{w_i}) = \left(\delta_{ss}(\mathbf{w_i}), \delta_{12}(\mathbf{w_i})\right)^\top$, where $\delta_{ss}(\mathbf{w_i})$ serves the reference level ($s = 1$ or 2), and one time-varying continuous z_{it} that is mapped to $g(z_{\mathbf{i}t})$ using L_2 distance**.** The beta-diversity $f_{\mathbf{i},t}$ was simulated based on

$$E\left\{f_{\mathbf{i},t} \mid t, \boldsymbol{\delta}(\mathbf{x}_{\mathbf{i}t}), \boldsymbol{\delta}(w_{\mathbf{i}}), g(z_{\mathbf{i}t}); \boldsymbol{\beta}\right\} = h_{\mathbf{i}t}(\boldsymbol{\beta}) = \exp\left(\mathbf{q}_{\mathbf{i}t}^\top \boldsymbol{\beta}\right), \ 1 \le t \le m. \tag{1}$$

For the response profile Edger (M1) where time is as a factor, we considered $m = 2$ and specified the null as
$\mathbf{q}_{\mathbf{i}t}^{M1} = (1, I_{\mathbf{i}2}(t), \delta_{12}(\mathbf{x_i}), \delta_{22}(\mathbf{x_i}), I_{\mathbf{i}2}(t) \cdot \delta_{12}(\mathbf{x_i}), I_{\mathbf{i}2}(t) \cdot \delta_{22}(\mathbf{x_i}), \delta_{12}(\mathbf{w_i}), g(z_{\mathbf{i}t}))$,
$\boldsymbol{\beta}_0^{M1} = \left(\beta_0, \beta_2^T, \beta_{12}^G, \beta_{22}^G, \beta_{2,12}^I, \beta_{2,22}^I, \eta_{12}, \xi_1\right)^\top = (0.0285, 0.7, 0.5, 0.8, 0.9, 1, 0.6, 0.4)^\top$.

For the semiparametric curve Edger (M2) with continuous time, we set $m = 3$ and
$\mathbf{q}_{\mathbf{i}t}^{M2} = (1, t, \delta_{12}(\mathbf{x_i}), \delta_{22}(\mathbf{x_i}), t \cdot \delta_{12}(\mathbf{x_i}), t \cdot \delta_{22}(\mathbf{x_i}), \delta_{12}(\mathbf{w_i}), g(z_{\mathbf{i}t}))$,
$\boldsymbol{\beta}_0^{M2} = \left(\beta_0, \beta_2^T, \beta_{12}^G, \beta_{22}^G, \beta_{12}^I, \beta_{22}^I, \eta_{12}, \xi_1\right)^\top = (0.0285, 0.7, 0.5, 0.8, 0.9, 1, 0.6, 0.4)^\top$.

2.1 | Complete Data

The explanatory variables for each subject was generated by $x_i \sim Bernoulli(p_x)$, $w_i \sim Bernoulli(p_w)$, $\mathbf{z}_i = (z_{i1}, ..., z_{im})^\top \sim N(\boldsymbol{\mu}_\mathbf{z}, \Sigma_\mathbf{z})$. We constructed their between-subject counterparts $\boldsymbol{\delta}(\mathbf{x_i})$, $\boldsymbol{\delta}(w_\mathbf{i})$ and $g(\mathbf{z}_{\mathbf{i}t})$ as described as above**.** We set $p_x = 0.5$, $p_w = 0.7$, $\boldsymbol{\mu}_\mathbf{z} = (5, 6)^\top$, $\Sigma_\mathbf{z} = diag(0.5, 0.6)$ under $m = 2$, and $\boldsymbol{\mu}_\mathbf{z} = (1, 2, 3)^\top$, $\Sigma_\mathbf{z} = diag(0.5, 0.6, 0.4)$ under $m = 3$.

To simulate Beta-diversity $f(\mathbf{y}_{\mathbf{i}t})$ that reflects the speficed null, covariates needed to be added to the *effect-free* Beta-diversity, which was created from a real study data. More specifically, we first simulated life-like OTUs based on their *empirical CDF* from the real data (using baseline OTU), we also used the *copula* to maximally preserve correlations among OTU features. Then we computed the Bray-Curtis Beta-diversity $d_{\mathbf{i}1}(\mathbf{y}_{\mathbf{i}1})$ from the simulated OTUs $\mathbf{y}_{\mathbf{i}1}$ at baseline. Next, we centered $d_{\mathbf{i}1}(\mathbf{y}_{\mathbf{i}1})$ with the true value of β_0 to create the "residual"

$$\varepsilon_\mathbf{i} = d_{\mathbf{i}1}(\mathbf{y}_{\mathbf{i}1}) - \beta_0,$$

as the *effect-free* Beta-diversity for the *reference* group at baseline.

Then we added group by time and covariate effects $\mathbf{q}_{\mathbf{i}t}^\top \boldsymbol{\beta}_0$ to create:

$$\widetilde{d}_{\mathbf{i}t}(\mathbf{y}_{\mathbf{i}t}) = \exp\left(\mathbf{q}_{\mathbf{i}t}^\top \boldsymbol{\beta}_0 \ + \ \varepsilon_\mathbf{i}\right) = \exp\left(\mathbf{q}_{\mathbf{i}t}^\top \boldsymbol{\beta}_0\right) \exp(\varepsilon_\mathbf{i}).$$

By setting $C_0 = E\left[\exp(\varepsilon_\mathbf{i})\right]$, we obtained simulated kernel response

$$f(\mathbf{y}_{\mathbf{i}t}) = C_0^{-1} \widetilde{d}_{\mathbf{i}t}(\mathbf{y}_{\mathbf{i}t}).$$

It is easily checked that $E\left[f(\mathbf{y}_{\mathbf{i}t}) \mid \mathbf{q}_{\mathbf{i}t}\right] = h(\mathbf{q}_{\mathbf{i}t}; \boldsymbol{\beta}_0) = \exp\left(\mathbf{q}_{\mathbf{i}t}^\top \boldsymbol{\beta}_0\right)$.

We estimated C_0 by the sample mean $C_0 = \binom{n}{2}^{-1} \sum_{\mathbf{i} \in C_2^n} \exp(\varepsilon_\mathbf{i})$ using a large $n = 5,000$, where $C_0 = 1.0001$ in our setting. $\beta_0 = 0.0285$ is estimated from the large sample with $\beta_0 = \binom{n}{2}^{-1} \sum_{\mathbf{i} \in C_2^n} d_{\mathbf{i}1}(\mathbf{y}_{\mathbf{i}1})$ for an independent sample of $n = 5,000$.

2.2 | Missing Data under Missing at Random (MAR)

Under M1, we let the transition probability from time 1 to 2 depend on $z_{\mathbf{i}1}$ (the time-varying continuous covariate at $t = 1$) with $\pi_{\mathbf{i},2}(\boldsymbol{\gamma}) = p_{\mathbf{i},2}(\boldsymbol{\gamma}_2) = \exp it(\gamma_{02} + \gamma_{z2} z_{\mathbf{i}1})$, where $\boldsymbol{\gamma} = \boldsymbol{\gamma}_2 = (\gamma_{02}, \gamma_{z2})^\top$ are the parameters for missingness. Under M2, the additional one-step transition probability from time 2 to 3 was specified as $p_{\mathbf{i},3}(\boldsymbol{\gamma}_3) = \exp it(\gamma_{03} + \gamma_{z3}^1 z_{\mathbf{i}1} + \gamma_{z3}^2 z_{\mathbf{i}2})$. Hence, $\pi_{\mathbf{i},2}(\boldsymbol{\gamma}) = p_{\mathbf{i},2}(\boldsymbol{\gamma}_2)$, $\pi_{\mathbf{i},3}(\boldsymbol{\gamma}) = p_{\mathbf{i},2}(\boldsymbol{\gamma}_2) p_{\mathbf{i},3}(\boldsymbol{\gamma}_3)$, where $\boldsymbol{\gamma} = \left(\boldsymbol{\gamma}_2^\top, \boldsymbol{\gamma}_3^\top\right)^\top$, and we set the truth $\boldsymbol{\gamma}_2 = (\gamma_{02}, \gamma_{z2})^\top = (0.1, 0.9)^\top$, $\boldsymbol{\gamma}_3 = \left(\gamma_{03}, \gamma_{z3}^1, \gamma_{z3}^2\right)^\top = (3, 0.7, 0.4)^\top$. To create missing responses, we further multiplied the complete data $f_{\mathbf{i},t} = f(\mathbf{y}_{\mathbf{i}t})$ by the simulated indicators $R_{\mathbf{i},t} \sim Bernoulli(\pi_{\mathbf{i},t}(\boldsymbol{\gamma}))$.

TABLE 1 Comparison of overall (omnibus) group difference on IBS study.

	Test Statistics (pseudo-F or Wald)	p-value
PERMANOVA	.617	.290
PERMDISP	10.497	.013
GLMM-MiRKAT	/	< .001
Edger	53.395	< .001

3 | COMPARISON ACROSS PERMDISP, PERMANOVA, GLMM-MIRKAT, AND THE PROPOSED APPROACH

3.1 | Target of Inference and Hypotheses

To clarify the conceptual distinctions among existing beta-diversity methods and the proposed framework, we include a summary comparison of PERMANOVA, PERMDISP, GLMM-MiRKAT, and the proposed Edger in Table 1 of main paper to highlight differences in targets of inference and hypotheses addressed.

PERMANOVA and PERMDISP are distance-based, permutation-driven methods that model a single beta-diversity matrix. PERMANOVA targets differences in centroid location of multivariate microbial compositions across groups, whereas PERMDISP targets differences in within-group dispersion (heterogeneity). Neither method is designed to accommodate longitudinal dependence or to explicitly model temporal trajectories; in practice, they are typically applied either at a single visit or to time-aggregated summaries.

GLMM-MiRKAT extends kernel-based regression to longitudinal settings by modeling host outcomes as a function of microbiome composition, with random effects accounting for within-subject correlation. Its target of inference is therefore the association between microbiome composition and host traits, rather than changes in microbial community structure.

In contrast, the proposed Edger framework treats beta-diversity itself as the response and directly models its evolution over time. The primary target of inference is change in community-level dissimilarity, allowing formal testing of time effects, group effects, and group-by-time interactions. This enables inference on ecological concepts such as convergence, divergence, and stabilization of microbial communities, which are not directly addressed by the other approaches.

3.2 | Additional Empirical Results for the Irritable Bowel Syndrome (IBS) Study

To further contextualize these distinctions, we conducted empirical comparisons using a real-world longitudinal microbiome dataset from an Irritable Bowel Syndrome (IBS) study with five repeated visits, as described in the main text. The original analysis of this dataset distinguished among the constipation-predominant (IBS-C), diarrhea-predominant (IBS-D), and healthy control (H) groups[1].

Here, we report empirical p-value comparisons for overall (omnibus) group differences using PERMANOVA and PERMDISP alongside GLMM-MiRKAT and the proposed Edger framework, with all analyses restricted to complete cases to ensure comparability. PERMANOVA and PERMDISP were applied to visit-aggregated beta-diversity, consistent with their standard cross-sectional usage, whereas GLMM-MiRKAT and Edger explicitly accounted for the longitudinal structure of the data.

As shown in Table 1, PERMANOVA did not detect a significant overall group difference, while PERMDISP identified significant differences in dispersion. Both GLMM-MiRKAT and Edger yielded highly significant results. Importantly, GLMM-MiRKAT tests for association between microbiome composition and IBS subtype but does not directly provide a unified test statistic for longitudinal community-level change. In contrast, Edgers omnibus test directly evaluates whether longitudinal trajectories of beta-diversity, which reflect that community-level heterogeneity dynamics differ across groups.

4 | DETAILS OF THE DIETARY INTERVENTION STUDY

Healthy adults (non-pregnant and non-lactating women and men, age 18-45 years) were recruited to a dietary study at Vanderbilt University Medical Center (VUMC), as described previously[2]. Briefly, individuals completed two dietary interventions periods

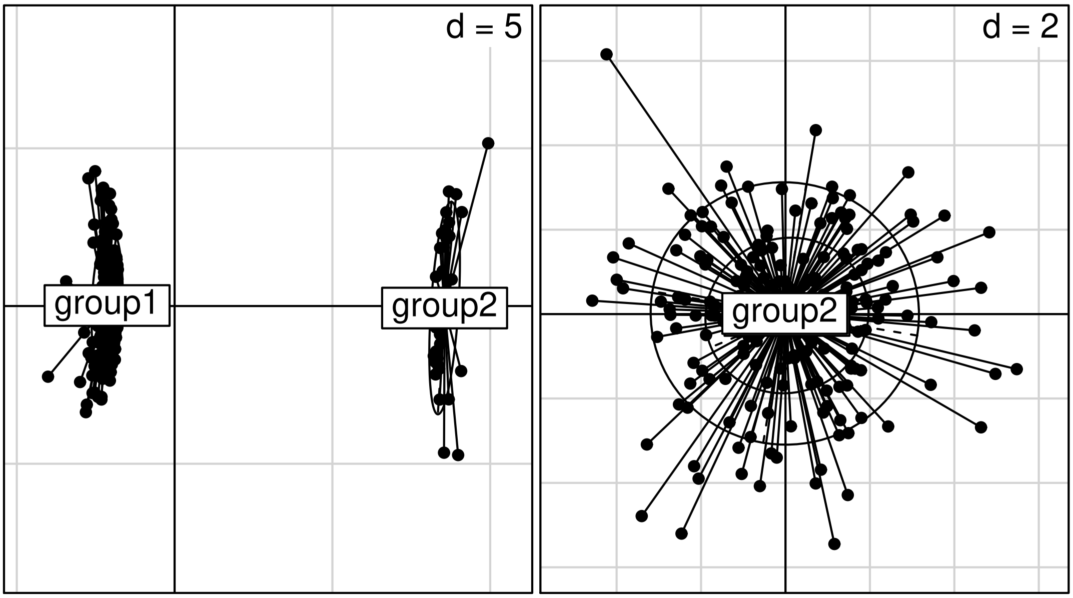

FIGURE 1 Examples demonstrating “location" (left) and “dispersion" (right) group differences using Principal coordinates analysis (PCoA).

TABLE 2 Simulation results for Response Profile (M1) and Continuous Time (M2) Edger under the null hypotheses using Jaccard beta-diversity.

	Type I Error					Time for one iteration (in *seconds*)				
	Edger		GLMM-MiRKAT			Edger	GLMM-MiRKAT			Default
Sample size	β^G_{22}	β^G_{12}	99	299	499		99	299	499	5000
100	.058	.062	.036	.042	.040	.104	.150	.326	.525	4.802
200	.048	.046	.038	.048	.054	.507	.570	1.125	1.674	14.028
400	.048	.050	.052	.052	.054	2.623	3.203	5.158	7.056	50.384
600	.048	.048	.038	.044	.040	7.309	9.986	14.167	18.201	111.894

of 7-days each, separated by 2-week washout, in a randomized cross-over design. Study menus were developed by research dietitians at the Vanderbilt Diet, Body Composition and Human Metabolism Core, and were designed to have an average daily macronutrient composition of 35% fat, 50% carbohydrate and 15% protein. The control diet contained ~3.5g/day lysine derived from foods. During the high-lysine diet, participants consumed the control diet, supplemented with 5g/day, L-lysine-HCL. Enrolled participants completed a total of 4 study visits at VUMC. Participants collected a stool sample within the 24 hours prior to each study visit (Commode Specimen Collection System, Fisher Scientific). Samples were maintained at 4°C, and aliquots made within 36 hours of sample collection. Aliquots were stored at -80°C prior to DNA isolation. All participants provided written, informed consent, and the study was approved by the Vanderbilt University Institutional Review Board. The study was registered at ClinicalTrials.gov, NCT04417218.

DNA was isolated from stool samples using the All Prep PowerFecal Pro DNA/RNA kit (Qiagen). Libraries were prepared and whole metagenome sequencing performed (150PE, ~10M reads/sample, NovaSeq 6000) at the Vanderbilt University Technologies for Advanced Genomics (VANTAGE) Core. DNA sequences in Fastq files were de-multiplexed, assembled, clustered, and phylogenetically classified using the Dragen metagenomic pipeline (BaseSpace, Illumina Inc).

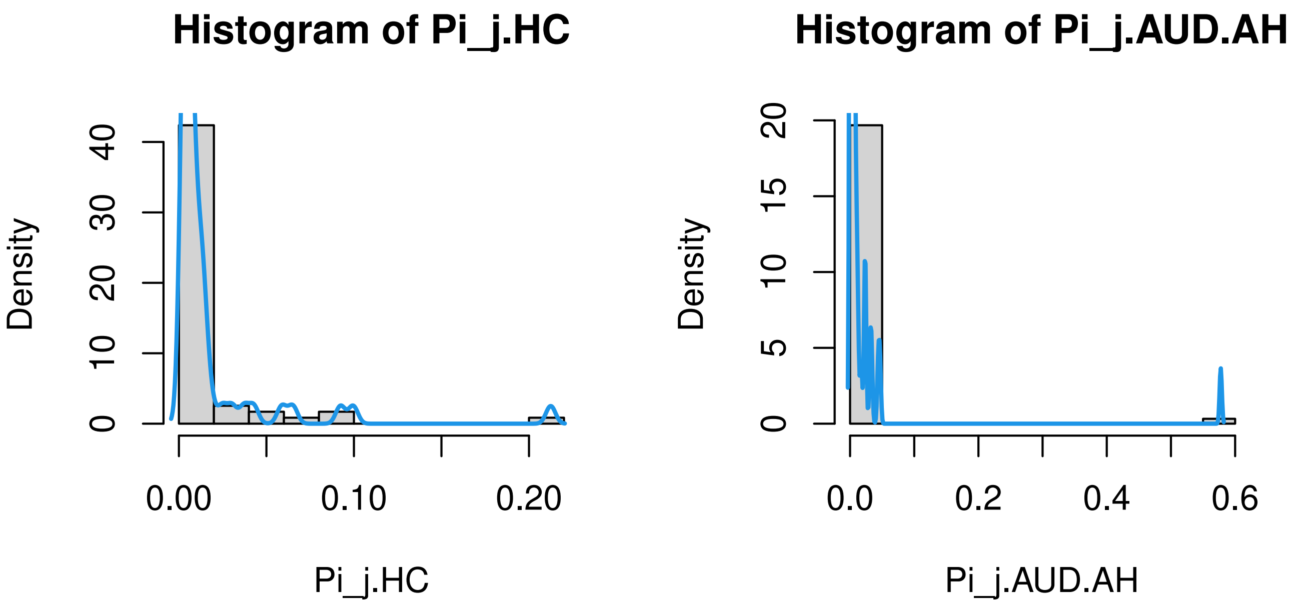

FIGURE 2 The histograms of microbiome OTUs from the real study data that contains two groups: HC vs. diseased (AUD.AH).

5 | SUPPLEMENTAL FIGURES

6 | SUPPLEMENTAL TABLES

REFERENCES

1. Mars RA, Yang Y, Ward T, et al. Longitudinal multi-omics reveals subset-specific mechanisms underlying irritable bowel syndrome. *Cell.* 2020;182(6):1460–1473.
2. Antonetti OR, Desine S, Smith HM, et al. The consumption of animal products is associated with plasma levels of alpha-aminoadipic acid (2-AAA). *Nutrition, Metabolism and Cardiovascular Diseases.* 2024;34:1496-1504. doi: 10.1016/j.numecd.2024.03.009

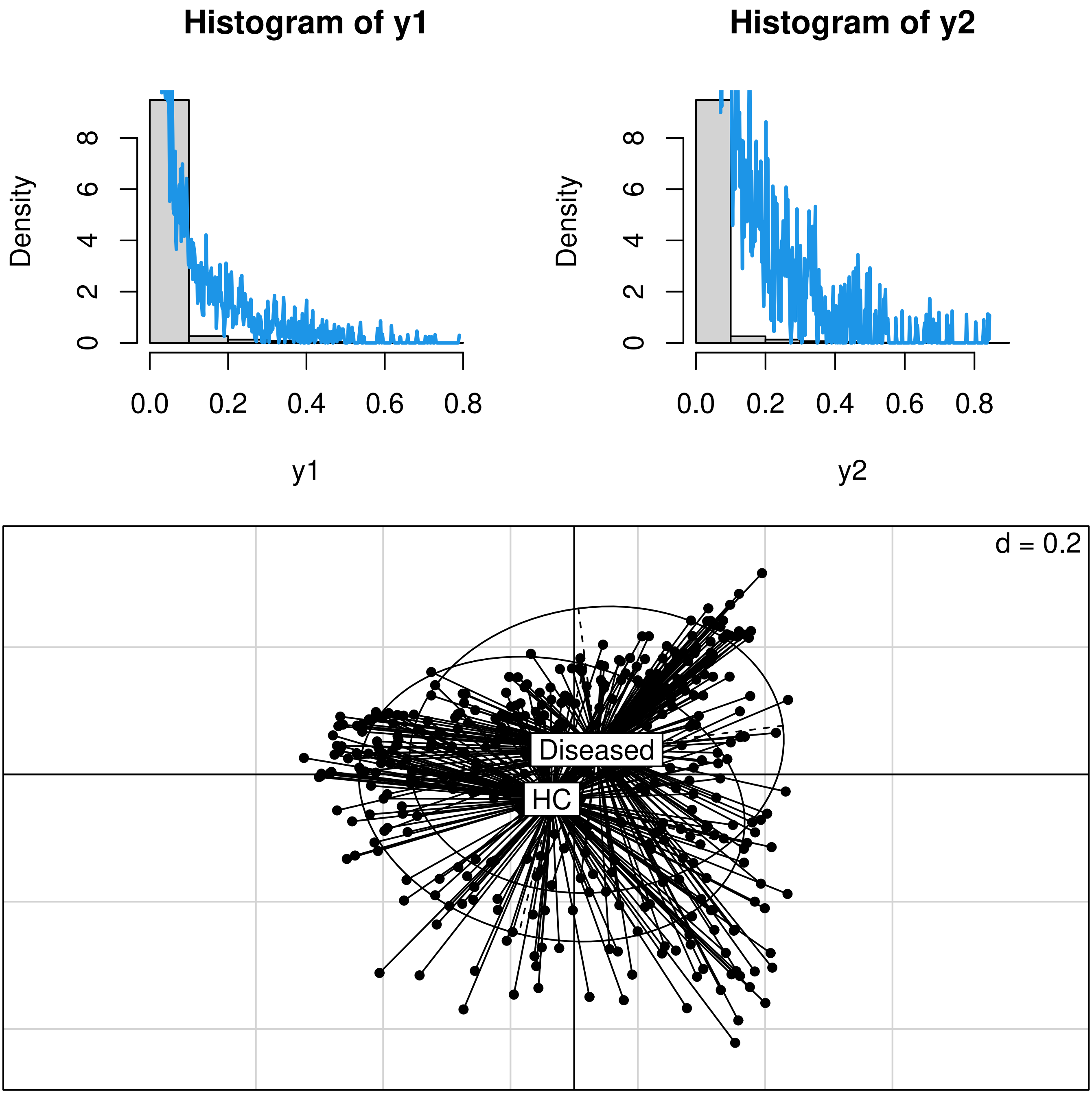

FIGURE 3 The histograms (a) and PCoA plot (b) of simulated OTUs for the two groups at $t = 1$ in case 1 to demonstrate their differences in location, or the centers.

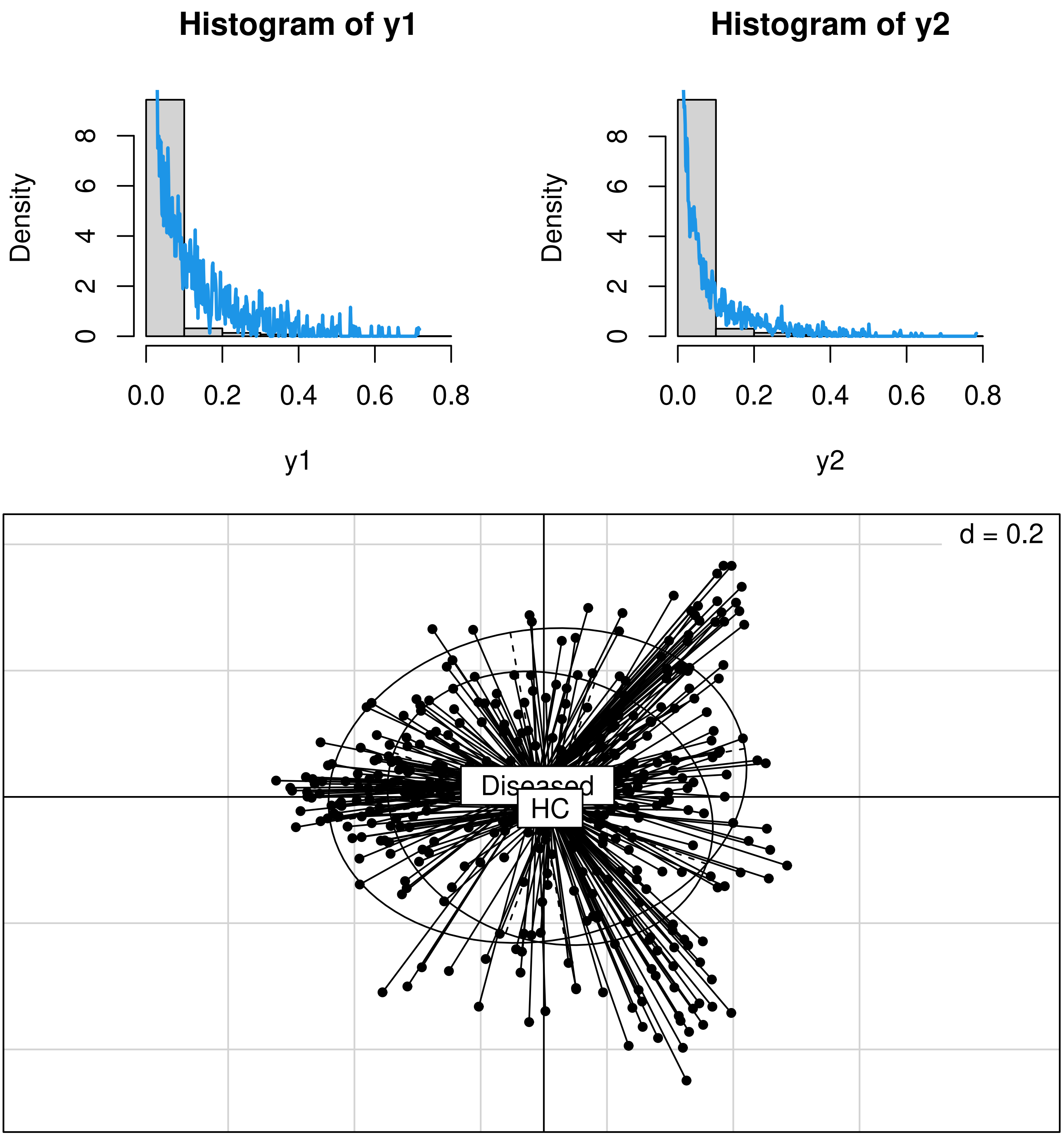

FIGURE 4 The histograms (a) and PCoA plot (b) of simulated OTUs for the two groups at $t = 1$ in case 2 to demonstrate their differences in variability, or dispersion.

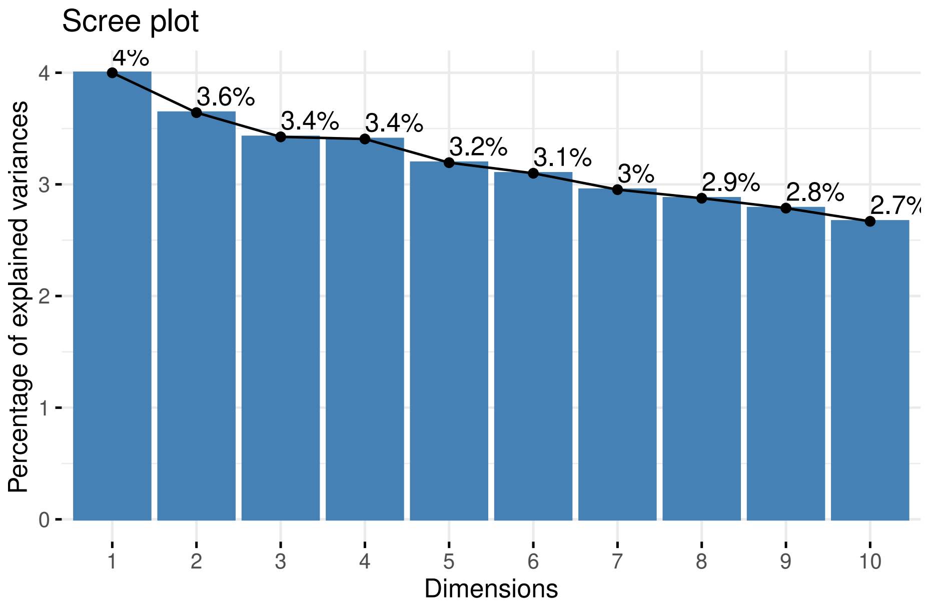

FIGURE 5 The scree plot of top 10 PCs from the simulated 100 covariates in case 3) of high-dimensional covariate adjustment, showing no definitive cut point from the PCA.

TABLE 3 Simulation comparisons of statistical power under the alternative: Edger (proposed) versus GLMM-MiRKAT (existing) using Jaccard beta-diversity.

"*Location*" difference				
	Edger	GLMM-MiRKAT		
Sample size	Omnibus	99	299	499
40	.476	.398	.428	.442
100	.992	.970	.976	.978
200	1	1	1	1
"*Location*" difference over time				
	Edger	GLMM-MiRKAT		
Sample size	Omnibus	99	299	499
40	.182	.024	.030	.036
100	.646	.032	.034	.030
200	.986	.030	.036	.038
250	.998	.040	.046	.038
300	1	.040	.038	.044
"*Scale*" difference				
	Edger	GLMM-MiRKAT		
Sample size	Omnibus	99	299	499
40	.424	.072	.092	.086
100	.860	.162	.178	.184
200	1	.546	.578	.588
"*Scale*" difference over time				
	Edger	GLMM-MiRKAT		
Sample size	Omnibus	99	299	499
40	.162	.028	.038	.034
100	.342	.040	.046	.052
200	.640	.032	.030	.036
250	.792	.040	.048	.042
300	.820	.048	.054	.050

TABLE 4 Empirical resampling study to evaluate Type I error under the null hypothesis using permutation of group labels of IBS study.

	Null Resampling (permuted labels)	
	GLMM-MiRKAT	Edger (omnibus)
Omnibus Group	0.049	0.048
